# Glycosaminoglycan biosynthesis pathway in host genome is associated with *Helicobacter pylori* infection

**DOI:** 10.1038/s41598-021-97790-7

**Published:** 2021-09-14

**Authors:** Dingxue Hu, Yueqi Lu, Daoming Wang, Chao Nie, Yan Li

**Affiliations:** 1 BGI-Shenzhen, Shenzhen, 518083 China; 2 College of Life Sciences, University of Chinese Academy of Sciences, Beijing, 100049 China

**Keywords:** Genetics, Microbiology, Gastroenterology

## Abstract

*Helicobacter pylori* is a causative pathogen of many gastric and extra-gastric diseases. It has infected about half of the global population. There were no genome-wide association studies (GWAS) for *H. pylori* infection conducted in Chinese population, who carried different and relatively homogenous strain of *H. pylori*. In this work, we performed SNP (single nucleotide polymorphism)-based, gene-based and pathway-based genome-wide association analyses to investigate the genetic basis of host susceptibility to *H. pylori* infection in 480 Chinese individuals. We also profiled the composition and function of the gut microbiota between *H. pylori* infection cases and controls. We found several genes and pathways associated with *H. pylori* infection (*P* < 0.05), replicated one previously reported SNP rs10004195 in *TLR1* gene region (*P* = 0.02). We also found that glycosaminoglycan biosynthesis related pathway was associated with both onset and progression of *H. pylori* infection. In the gut microbiome association study, we identified 2 species, 3 genera and several pathways had differential abundance between *H. pylori* infected cases and controls. This paper is the first GWAS for *H. pylori* infection in Chinese population, and we combined the genetic and microbial data to comprehensively discuss the basis of host susceptibility to *H. pylori* infection.

## Introduction

More than half of the world population were infected by *Helicobacter pylori*^1^*.* It is the major cause of gastritis (80%) and gastroduodenal ulcer disease (15–20%) and the only bacterial pathogen believed to cause gastric cancer^2,3^. The prevalence of *H. pylori* infection has large amounts of variations among countries. One of the causes may be that *H. pylori* populations are very diverse, due to point mutations and larger substitutions, insertions or deletions that may involve one or more genes or multigene segments (including restriction/modification genes and at least one pathogenicity island). Divergence among *H. pylori* strains would be further increased by differences among people in traits important to individual strains^4^. Numerous epidemiological studies have investigated the associations between polymorphic genetic elements of *H. pylori* and gastric disease development^5,6^. One study found that gastric cancer was associated with the presence of *babA* and genes in the *cag* pathogenicity island of *H. pylori* through comparing the strains isolated from gastric cancer patients and gastritis individuals^5^.

In addition to the variation of *H. pylori* genome, the host genetic variation also contributed to the *H. pylori* susceptibility^7,8^, the reported heritability was 57%^8^. So far, only one genome-wide association study (GWAS) of *H. pylori* infection was performed in European population^9^. Genetic variations in the toll-like receptor (TLR) locus were found to be associated with *H. pylori* seroprevalence. TLRs are important pattern recognition receptors on cellular surfaces in the innate immune system^10^, known to be essential in providing protective immunity against infection. Many studies reported that TLRs were associated with *H. pylori* infection^11,12^. On the *H. pylori* side, the immune response is triggered by bacterial membrane components, including lipopolysaccharides and lipid A, as well as cytotoxins and *H. pylori* urease activity^13^. Several genetic studies have demonstrated that the variants in *IL1B* were associated with susceptibility to gastric cancer among *H. pylori* infected individuals, this suggested that immune-related genes may be involved in *H. pylori* infection^14,15^. However, there is no GWAS for *H. pylori* infection on Chinese population.

On the other hand, gut microbiota was reported to play a role in regulating host metabolic function, protecting against pathogen infections and promoting immune system maturation. Previous studies reported some alterations of gut microbiota in *H. pylori* infected patients^16,17^. But there is a lack of research putting genetic susceptibilities and gut microbiome study together to integrate more information for understanding the mechanism of *H. pylori* infection.

Therefore, we carried out a GWAS on *H. pylori* infection and also compared the profiles of gut microbiota between cases and controls with the aim of identifying genetic variants influencing *H. pylori* infection and gaining more insights into the mechanism of *H. pylori* in Chinese population.

## Results

### Study sample

224 *H. pylori* infected positive individuals and 256 negative individuals were selected from an existing data set with the results of carbon-13 urea breath test (C13 test) for *H. pylori* infection. The written form of consent was signed for each individual. The C13 test results include diagnosis to *H. pylori* infection and the delta over baseline (DOB) values for cases. The distribution of DOB values for cases was shown in Supplementary Fig. S1. The detailed characteristic statistics of the samples were described in Table 1. The case and control groups were designed to match age, sex and body mass index (BMI). There were no significant differences between two groups in age, sex and BMI.

**Table 1:** Characteristic statistics of the study population.

	Number (N)	Sex		Age		BMI	
		female count (percent)	Male count (percent)	Mean (SD)	Range	Mean (SD)	Range
Case	224	127 (56.70%)	97 (43.30%)	30.78 (5.46)	22–58	22.01 (3.00)	15.20–30.60
Control	256	136 (53.13%)	120 (46.88%)	31.18 (5.40)	22–69	22.25 (3.22)	15.70–37.80
Total	480	263 (54.79%)	217 (45.21%)	30.99 (5.46)	22–69	22.14 (3.12)	15.20–37.80

After Wilcoxon test, there was no significance in age difference between case and control. In the logistic regression model, *H. pylori* infection status ~ sex, we found there was no significant correlation between sex and *H. pylori* infection. BMI had no significant difference in the cases and controls.

### Association analysis on *H. pylori* infection status and DOB measurements

**Figure 1:**
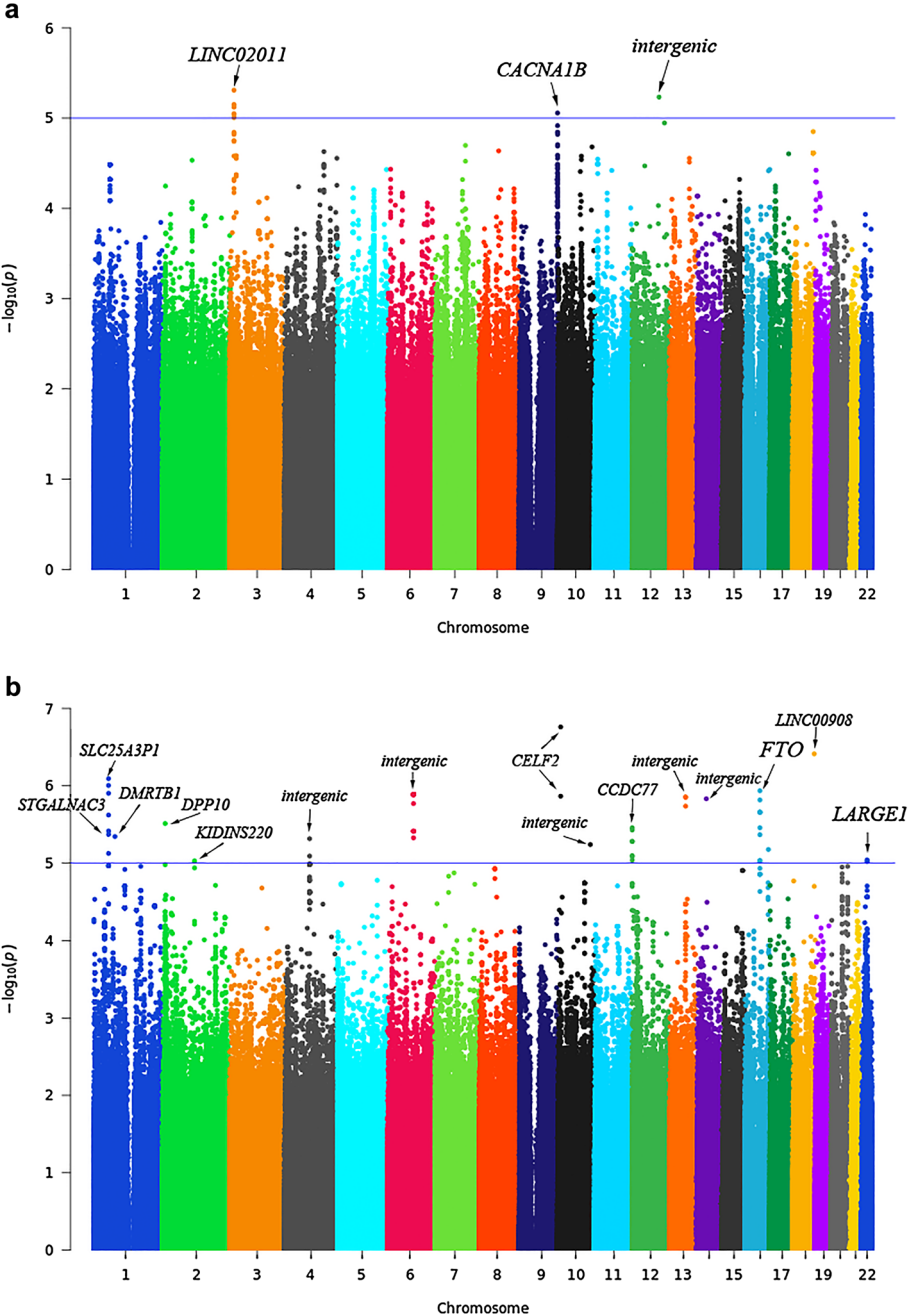
Manhattan plots of genome-wide association analyses. (**a**) Manhattan plots presenting the − log_10_ *P* values from the GWAS adjusted for the top two principal components of the *H. pylori* infection cases versus all controls and (**b**) the DOB values in *H. pylori* infection cases. The blue line indicates the threshold for suggestive significant association (*P* < 1e−5). The genes that suggestive significant variants encoded are marked in the plot.

In order to identify genetic loci involved in *H. pylori* infection, we constructed two models in GWAS after standard quality control (QC) processes. Logistic regression was performed in 224 cases and 256 controls and linear regression was performed in 224 cases. The scree plot of principal component analysis (PCA) was shown in Supplementary Fig. S2a. The top 2 components could explain most of the variations of the population and no clear population stratification was observed (Supplementary Fig. S2b). The variant-based associations showed no genome-wide significant single nucleotide polymorphisms (SNPs) (*P* < 5e−8). There were still SNPs reached suggestive level (*P* < 1e−5) (Fig. 1), 10 SNPs for logistic regression and 59 SNPs for linear regression (Supplementary Table S1 and Supplementary Table S2). The Q–Q plots of both associations were shown in Supplementary Fig. S3. The inflation factor was 1.00445 for case/control analysis and 1.01076 for DOB values association, indicating there were no population stratification for the analyses.

**Figure 2:**
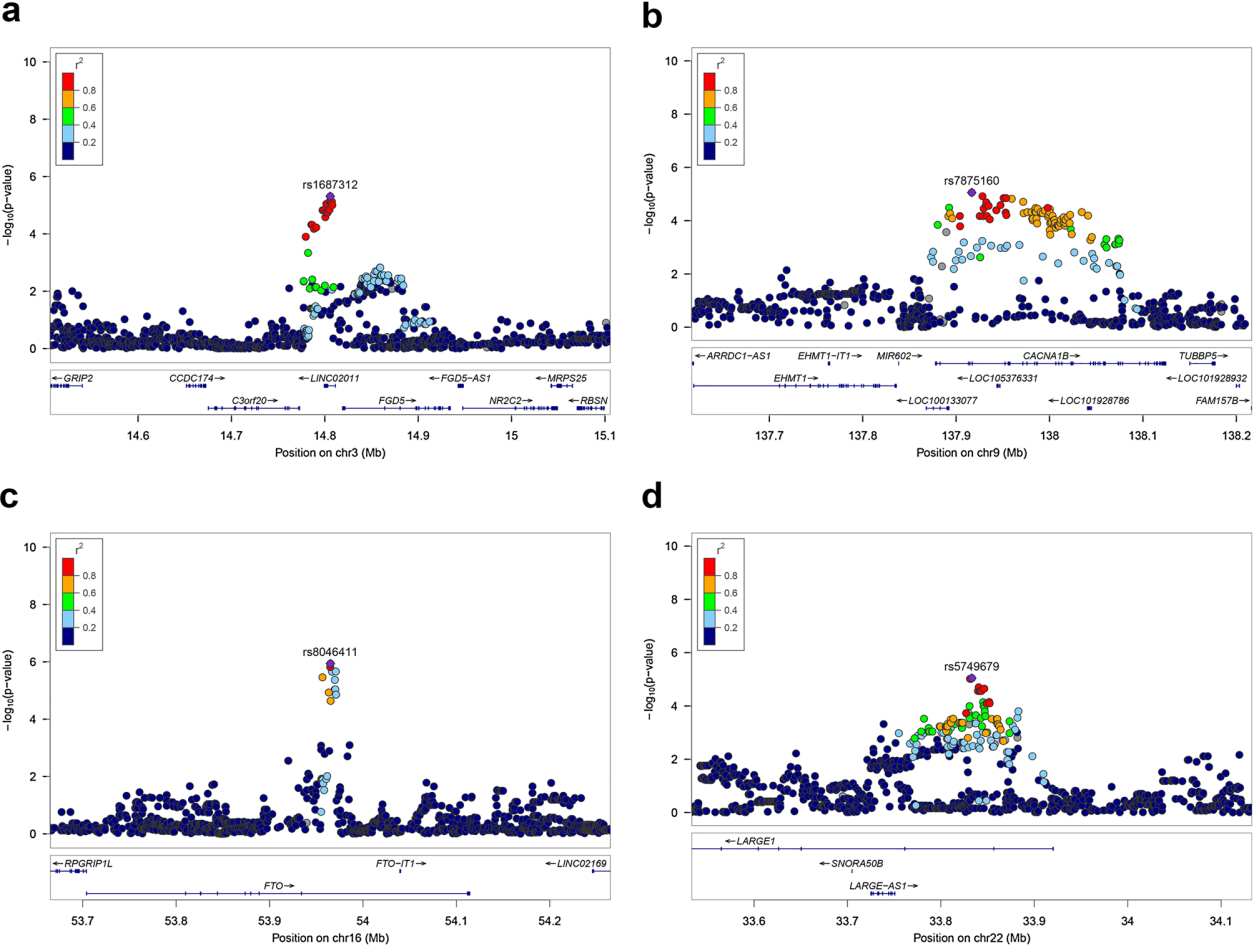
Regional association plots for the regions containing associated genes. Regional association plots for the *LINC02011* (**a**), *CACNA1B* (**b**), *FTO* (**c**) and *LARGE1* (**d**). *LINC02011* (**a**) and *CACNA1B* (**b**) were identified based on the GWAS results from the *H. pylori* infection cases versus controls, *FTO* (**c**) and *LARGE1* (**d**) were identified based on the DOB values association study of *H. pylori* infection cases. The color of the variants is based on the linkage disequilibrium with rs1687312 (*LINC02011*) (**a**), rs7875160 (*CACNA1B*) (**b**), rs8046411 (*FTO*) (**c**) and rs5749679 (*LARGE1*) (**d**). For all plots, each point represents a SNP, where the x axis represents the position of the SNPs and the y axis represents the − log10 *P* values of the GWAS results. Each point is color-coded with the r^2^ value as calculated with the source of LD information retrieved from hg38/1000 Genomes Nov 2014 EAS (Asian). Plots showed the most significant SNPs flank 300 kb.

Then we further investigated the regions harbor suggestive significant SNPs. In case/control association, two signals were located in genes *LINC02011* (Fig. 2a) and *CACNA1B* (Fig. 2b) regions, another signal was located in intergenic region between *NEDD1* and *RMST* (Supplementary Table S1 and Supplementary Fig. S4). Copy number variation in *LINC02011* region has been reported to be associated with autism spectrum disorder^18,19^. This region also harbored another non-coding gene *FGD5-AS1,* it was reported to regulate gastric cancer cell proliferation and chemoresistance^20^. Protein encoded by gene *CACNA1B* was involved in calcium channel activity. This gene has been found recurrently mutated in 2 or more tumor tissues of 5 gastric cancer patients with *H. pylori* infection^21^. In linear regression based GWAS, 59 SNPs passed the nominal significance level (*P* < 1e−5). The signals were located in genes *SLC25A3P1, DMRTB1, ST6GALNAC3, KIDINS220, DPP10, CELF2, CCDC77, FTO, ATP2C2-AS1* and *LARGE1* regions and some intergenic regions. Their summary statistics were shown in Supplementary Table S2 and Supplementary Fig. S5. Among these regions, *FTO* (Fig. 2c) was strongly associated with obesity and BMI^22^. Obesity has been reported to be associated with *H. pylori* infection^23^. It is also a risk factor for gastric cancer^24^. One study found that gastric cancer tissues had high *FTO* expression as compare with the adjacent non-cancerous tissues^25^. *LARGE1* gene (Fig. 2d) was involved in immune response for disease pathogens^26^.

### Set-based association for further identification of susceptibility genes and pathways for *H. pylori* infection

To improve GWAS power, set-based analyses were performed, which combined the GWAS summary statistics of all variants within a putative biologically functional unit (coding region and possible regulatory region, genes within one pathway) to obtain a single *P* value that represented the significance level of the associations between *H. pylori* infection and the functional unit. Our SNPs were mapped onto 25,928 genes for gene-based association. The top associated genes for case/control comparison were *LOC105376331* and *LINC02011* (gene-level *P* < 1e−04). And the most significant genes for linear regression were *LARGE1* and *LINC00561* (gene-level *P* < 1e−04). They failed to pass genome-wide gene-based significance threshold (0.05/25928). Then we further mapped these genes to canonical pathways from molecular signature database^27,28^.

164 pathways were included in the analyses. Top pathways associated with *H. pylori* infection (*P* < 0.05) were listed in Tables 2 and 3. In the logistic model-based pathway association, the glycosphingolipid biosynthesis related pathway (glycosphingolipid biosynthesis—lacto and neolacto series) was the most significant pathway. One study reported that after *H. pylori* eradicated successfully, glycosphingolipid biosynthesis—lacto and neolacto series pathway was significantly altered^29^. In the linear model-based pathway association, the most significant pathway was antigen processing and presentation pathway. Glycosaminoglycan biosynthesis of heparan sulfate (HS) pathway was significant in the both two models, it was a pathway correlated with bacterial adherence^30^. Several immune-related pathways were ranked top in the pathway-based association, indicating the variation of immune systems leading to different infection status of *H. pylori* infection. Folate biosynthesis pathway was also significantly correlated with different infection status of *H. pylori* infection. Study reported that folate level was significantly decreased (18.6%) in *H. pylori* infected individuals than those who were not infected^31^.

**Table 2:** Candidate pathways associated with *H. pylori* infection in logistic regression model.

Pathway set	No.SNPs	Chisq(Obs)	*P* value	TopSNP.Pvalue	TopSNP
KEGG_GLYCOSPHINGOLIPID_BIOSYNTHESIS_LACTO_AND_NEOLACTO_SERIES	18	58.7025	2.03E−02	9.41E−03	rs872451
KEGG_GLYCOSAMINOGLYCAN_BIOSYNTHESIS_HEPARAN_SULFATE	80	149.922	2.50E−02	3.82E−03	rs7760512
KEGG_CYTOSOLIC_DNA_SENSING_PATHWAY	14	37.2182	3.88E−02	7.01E−03	rs118156647
KEGG_AXON_GUIDANCE	120	207.521	4.33E−02	1.90E−03	rs12572571
KEGG_NATURAL_KILLER_CELL_MEDIATED_CYTOTOXICITY	25	58.616	4.68E−02	6.96E−03	rs13420683
KEGG_PRIMARY_IMMUNODEFICIENCY	25	58.616	4.68E−02	6.96E−03	rs13420683
KEGG_FC_GAMMA_R_MEDIATED_PHAGOCYTOSIS	4	10.6895	4.90E−02	3.88E−02	rs57919378
KEGG_RNA_DEGRADATION	16	37.3961	4.96E−02	6.68E−03	rs34113220

No.SNPs: number of SNPs in the pathway; Chisq(Obs): sum of chi-squared test-statistics of all SNPs in the set; *P* value: pathway set-based test *P* value; TopSNP.Pvalue: smallest single-SNP GWAS *P* value in the pathway set; TopSNP: the top associated GWAS SNP. The rsID is based on dbSNP build 138.

**Table 3:** Candidate pathways associated with *H. pylori* infection in linear regression model.

Pathway set	No.SNPs	Chisq(Obs)	*P* value	TopSNP.Pvalue	TopSNP
KEGG_ANTIGEN_PROCESSING_AND_PRESENTATION	46	126.12	9.74E−03	1.03E−03	rs12204338
KEGG_AUTOIMMUNE_THYROID_DISEASE	46	126.12	9.74E−03	1.03E−03	rs12204338
KEGG_HEDGEHOG_SIGNALING_PATHWAY	6	23.2668	1.60E−02	5.35E−03	rs9576171
KEGG_TERPENOID_BACKBONE_BIOSYNTHESIS	9	32.6463	1.91E−02	8.99E−03	rs61836189
KEGG_NEUROACTIVE_LIGAND_RECEPTOR_INTERACTION	29	70.052	2.28E−02	1.56E−03	rs2146490
KEGG_ADIPOCYTOKINE_SIGNALING_PATHWAY	19	48.4956	2.97E−02	2.09E−02	rs12602449
KEGG_JAK_STAT_SIGNALING_PATHWAY	19	48.4956	2.97E−02	2.09E−02	rs12602449
KEGG_FOLATE_BIOSYNTHESIS	21	46.2834	4.66E−02	1.16E−02	rs13131273
KEGG_GLYCOSAMINOGLYCAN_BIOSYNTHESIS_HEPARAN_SULFATE	80	135.496	4.77E−02	5.04E−03	rs12195272

No.SNPs: number of SNPs in the pathway; Chisq(Obs): sum of chi-squared test-statistics of all SNPs in the set; *P* value: pathway set-based test *P* value; TopSNP.Pvalue: smallest single-SNP GWAS *P* value in the pathway set; TopSNP: the top associated GWAS SNP. The rsID is based on dbSNP build 138.

### Replication of previous reported *H. pylori* infection related genes

We have checked the two signals reported in previous GWAS in European population^9^. The rs10004195 in gene *TLR1* was replicated in the linear association study (*P* = 0.02), of which the minor allele T has reduced effects on *H. pylori* infection. The SNP rs368433 was filtered out by our QC analysis because it is a low-frequency SNP in Asian (dbSNP: minor allele frequency (MAF) = 0.009). The SNP rs10004195 was located in a region which included multiple *TLRs* (*TLR10*, *TLR1* and *TLR6*). *TLRs* play crucial roles in activating innate immunity. The SNP rs368433 was located in *FCGR2A* gene region. Further gene-based association combining the information from neighboring SNPs within a region showed that *TLRs* region was replicated (*P* < 0.05) based on the linear regression results. *FCGR2A* was not replicated (*P* > 0.05).

### Differences of gut microbiomes were identified between *H. pylori* infected groups and controls

Stool samples from 403 individuals were collected and metagenome sequencing was utilized to study the gut microbiome between 185 *H. pylori* infected cases and 218 controls. In total, we identified 961 taxonomic categories from kingdom to species assigned by MetaPhlAn2^32^. Among them, 782 categories were classified to the kingdom of bacteria and its sub-levels. After removing categories with carriers less than 10%, 301 categories in different taxonomic levels were remained.

Then we compared the differences of bacterial abundance between case and control groups at different taxonomic levels. After false discovery rate (FDR) multiple testing adjustment, the abundance of two species were significantly different, *Holdemanella biformis* (FDR-adjusted *P* = 0.0579) and *Catenibacterium mitsuokai* (FDR-adjusted *P* = 0.0785) (Table 4). These 2 bacteria categories all demonstrated higher presence ratio in case group rather than control group. *H. biformis* is one of the species in the family *Erysipelotrichaceae* which is a key butyrate producing taxa^33–35^. The *Erysipelotrichaceae* was associated with inflammation-related gastrointestinal diseases^36^. As for *C. mitsuokai*, its abundance would increase in mice after intervention with a high-fat and high-sugar diet^37^, and it was closely related to unhealthy fasting serum lipid profile in obese women^38^. This species is the only known species from the genus *Catenibacterium*. The abundance of three genera were significantly different, ‘*Erysipelotrichaceae noname*’, (FDR-adjusted *P* = 0.0212), *Catenibacterium* (FDR-adjusted *P* = 0.0349), *Prevotella* (FDR-adjusted *P* = 0.0861) (Table 4). ‘*Erysipelotrichaceae noname*’ is an unidentified genus annotated by MetaPhlAn2^32^ below the family *Erysipelotrichaceae* mentioned above. *Prevotella* is one of the bacteria producing acetate while acetic acid participating in cholesterol synthesis^39,40^. The abundance of these categories were higher in the *H. pylori* infected groups. We didn’t observe significant differences between *H. pylori* infected cases and controls in terms of alpha and beta diversities of bacteria. The species classified below these three genera were listed in Supplementary Table S3.

**Table 4:** Bacteria associated with *H. pylori* infection in Wilcoxon test.

Taxo	Mean difference	*P* value	FDR-adjusted P	Presence ratio	
				HPI negative	HPI positive
*Holdemanella biformis*	0.0008	0.0004	0.0579	0.2156	0.3838
*Catenibacterium mitsuokai*	0.0002	0.0010	0.0785	0.0596	0.1622
*‘Erysipelotrichaceae noname’*	0.0008	0.0003	0.0212	0.4266	0.5946
*Catenibacterium*	0.0002	0.0010	0.0349	0.0596	0.1622
*Prevotella*	0.0648	0.0037	0.0861	0.8716	0.8973

Taxo: identified bacteria names at last taxonomic level by MetaPhlAn2; Mean difference: difference of bacteria abundance between *H. pylori* infection positive group and *H. pylori* infection negative group; *P* value: *P* value of Wilcoxon test; FDR: taxonomic level-based FDR adjusted *P* value; Presence ratio: ratio of non-zero abundance individuals to all people, HPI negative: *H. pylori* infection negative; HPI positive: *H. pylori* infection positive.

Furthermore, we identified 37 *H. pylori* infection specific metabolic pathways which have different abundance in the case and control groups through logistic regression at nominal significance level (*P* < 0.05) (Supplementary Table S4). These results suggested that pathways involved in amino acids biosynthesis (e.g.: L-isoleucine, L-ornithine were observed in *H. pylori* infection negative individuals, while L-lysine, L-lysine biosynthesis, S-adenosyl-L-methionine cycle I, L-citrulline metabolism and L-histidine degradation pathways had higher abundance in *H. pylori* infection positive groups), nucleotides metabolism (e.g.: pyrimidine related pathways were enriched in *H. pylori* infection negative group, while purine related pathways were enriched in *H. pylori* infection positive groups.), and other energy metabolites (e.g.: pyridoxal 5'-phosphate biosynthesis, glycolysis, homolactic fermentation, mannitol cycle, folate related pathways and peptidoglycan pathways) may be different between *H. pylori* infection case and control groups. Interestingly, we found that folate biosynthesis pathway was associated with *H. pylori* infection in both our genetic and microbiome study. It has been reported that folate level was significantly decreased (18.6%) in *H. pylori* infection individuals than those who were not infected in a low-income population in the United States^31^.

## Discussion

In this study, we performed genome-wide association study on 224 *H. pylori* infected individuals and 256 negative individuals and also compared their differences of gut microbiome with the aim of gaining more insights into the bases and consequences of *H. pylori* infection. We identified 3 genetic regions which were associated with *H. pylori* infection status and 14 genetic regions which were related to the bacteria load at suggestive significance level (*P* < 1e−5). Further pathway-based analysis showed that several pathways were associated with *H. pylori* infection. Those pathways were related to immune response and glycosylation. One of the two reported loci from previous GWAS analysis was replicated in our data. In gut microbiome analysis, 2 species, 3 genera and several pathways were identified to have different abundance between *H. pylori* infected cases and controls.

While comparing the results with the reported GWAS about *H. pylori* infection in 2013^9^, we found that rs10004195 in gene *TLR1* was associated with the severity of *H. pylori* infection (*P* = 0.02023), but it was not associated with the infection status. TLRs are known to be essential in providing protective immunity against infection. The minor allele of rs10004195 may be associated with less efficiency of anti-inflammatory TLR1–TLR2 signaling^9^. The other reported SNP rs368433 was filtered out by QC in our genetic data due to its low frequency in Chinese population (MAF = 0.009).

One possible cause of these different results may be the distinct methods for identifying *H. pylori* infection. In our study, we adopted C13 test to detect *H. pylori* infection. While in the GWAS for European population^9^, they tested anti-*H. pylori* serum IgG antibody titers. C13 test has higher sensitivity and specificity than anti-*H. pylori* serum IgG antibody titers test^41^. Although, anti-*H. pylori* serum IgG antibody titers could represent previous infection, it can hardly represent the current bacteria load in an individual. Besides, C13 test is a more convenient handling method. Therefore, we have chosen C13 breath tests in our study to measure both onset and severity of *H. pylori* infection. In addition, the population ancestry differences could be another cause for the variation of GWAS results. SNP rs368433 is common in European population and significantly associated with *H. pylori* infection^9^. However, it is a low frequency SNP (MAF = 0.009) in Chinese population, and it was filtered out in our QC processes. We also checked the adjacent SNPs whose position are close to rs368433, they were not associated with *H. pylori* infection in our data. The true causal variant could still be hidden in this genomic region, but due to different linkage disequilibrium (LD) structure among European and Chinese populations, the alleles tagging true causal variants may be different. Future fine mapping will be required to extend the region and dig the hidden information.

Another difference for *H. pylori* infection between Chinese and European population is that they carry distinct *H. pylori* strains^4^. Almost all Asian *H. pylori* strains contain the complete *cag*-pathogenicity island (PAI) which was associated with more severe disease in *H. pylori* infected individuals^4^.

When comparing our results to existing functional studies, the functions of our newly identified genes and pathways were highly relevant to *H. pylori* infection. *FGD5-AS1* regulates cancer gastric cell proliferation^20^. *CACNA1B* had accumulated somatic mutations in gastric tumor with *H. pylori* infection^21^. Genetic variants associated with obesity were also ranked top in our results. *FTO* is one of the leading genes associated with obesity and BMI^42,43^. Studies also found that gastric cancer tissues had high *FTO* expression compared with the adjacent non-cancerous tissues^25^. Obesity was considered as a risk factor for *H. pylori* infection^44^. Interestingly, differences of obesity related bacteria were also identified in our gut microbiome analysis. *C. mitsuokai* was enriched in *H. pylori* infection positive group, which was also enriched in obese women^38^. The associations among obesity, genetics of obesity, obesity related bacteria and *H. pylori* infection were reported pair wisely, but their causal relationships are still largely unknown. It is reported that *H. pylori* persistent infection had a negative effect on the fall of BMI values in Chinese obese population^45^. This comorbidity between *H. pylori* infection and obesity may be caused by the pleiotropic effects of *FTO* and other genes. We also found that glycometabolism-related genes and pathways were associated with *H. pylori* infection. *LARGE1* encoded a glycosylase, which played an important role in host–pathogen interactions^46^. Our pathway-based associations identified that glycosphingolipid biosynthesis and glycosaminoglycan biosynthesis were associated with *H. pylori* infection. Glycosphingolipids (GSLs) are components of cell-surface or basement membrane-associated proteoglycans produced by both epithelial and mesenchymal cells^47^. They are known to play a role as receptors in pathogen invasion^48^. Glycosaminoglycans have been shown to be recognized by *H. pylori* outer-membrane proteins and to be involved in the adhesion of the bacteria in cell-line models^30^. In the functional pathway enrichment of microbiome association study, we found that glycolysis related pathways were correlated with *H. pylori* infection. Many studies found that *H. pylori* can metabolize glucose by both oxidative and fermentative pathways^49–53^. *H. pylori* infected gastric epithelial cells have exhibited increased glycolysis and increased expression of Lon protease 1 (Lonp1), Lonp1 is a key regulator of metabolic reprogramming in *H. pylori*-induced gastric carcinogenesis^54,55^. We also identified two immune-related pathways, including antigen-processing and presentation pathway as well as autoimmune thyroid disease pathway. Study has reported that *H. pylori* affects the antigen presentation activity through microRNA^56^. *H. pylori* infection also has immune evasion strategies. Therefore, it was as expected that the genetic variations of immune-related genes may affect the susceptibilities of *H. pylori* infection.

While extensive number of studies have been focused on the alterations of gastric microbiota^29^ since *H. pylori* mainly colonizes the stomach^57^, little is known about the impact of *H. pylori* on gut microbiota. We have performed association analyses between *H. pylori* infection status and gut microbiome using shotgun meta-genome sequencing. Compared to existing 16S rRNA gene sequencing for gut microbiota, shotgun sequencing has more power to identify less abundant taxa than 16S sequencing^58^. We identified 2 species, 3 genera and several pathways had differential abundance between *H. pylori* infected cases and controls. Interestingly, we found that folate biosynthesis pathway was associated with *H. pylori* infection in both genetic and microbiome studies. Reduction of vitamin B12 and folate concentrations has been reported in *H. pylori*-infected patients^59^. Folate acid was synthesized by bacteria^60^. Altogether, it is suggested that *H. pylori* infection could alter gut microbiome and thereby influence the nutrition supply of the host. More metabolomic and nutrition studies may be required to study the consequences of *H. pylori* infection.

There are some limitations of current study. The sample size was not large enough to boost the signals to genome-wide significance. We have leveraged on the set-based association by combining the SNPs within a biological meaningful set and generated a summed statistic for the whole set. Our set-based association identified glycosaminoglycan and immune-related pathways, which were functional relevant with *H. pylori* infection. We also studied the gut microbiome differences between *H. pylori* infected cases and controls, the results showed more supporting evidence that the glycosylation related function was involved in *H. pylori* infection. Together, we provided a list of candidate genes, pathways and bacteria associated with *H. pylori* infection for further replication and validation.

## Methods

### Sample recruitment and data collection

The samples of current study were selected from a cohort study of Shenzhen local area. Individuals who had C13 test and no symptoms of any gastrointestinal conditions were selected. C13 test is used for detecting *H. pylori* infection. It involves taking C13 isotope-labeled urea molecules by mouth, and then measuring the changes in carbon dioxide in the exhaled carbon by an isotope mass spectrometer. The main measurement index was the δ value, which measured the ^13^C/^12^C ratio in the breath samples. DOB value is the difference between post-drug δ value minus pre-drug δ value. *H. pylori* infection is considered positive if DOB ≥ 4. Otherwise, *H. pylori* infection is negative (DOB < 4). Individuals who were diagnosed as *H. pylori* infection for both year 2017 and 2018 were further considered as the cases in our study. Individuals with DOB value equal to 0 were defined as control to ensure negative infection. At the end, 480 subjects (female N = 263, male N = 217) were selected for the association analyses.

### Whole genome sequencing

The whole blood drawn from the participant vein was stored in the ethylenediaminetetraacetic acid (EDTA) anticoagulant tubes to avoid hemolysis, while the plasma was obtained by centrifugation (3000 rpm, 10 min) and was preserved at – 80 ℃ until assay. The white cells were isolated for genomic DNA extraction. Whole-genome sequencing (WGS) to 30× were conducted on BGI-seq500 sequencer. WGS data were aligned and variants called by the Picard/BWA^61^/GATK^62^ pipeline. SNPs with mapping quality greater than 40, sequencing depth greater than 4, variant quality greater than 2.0, Phred score of Fisher’s test *P* value for stand bias smaller than 60.0, haplotype score smaller than 13.0 and distance of alternative allele from the end of reads greater than 8.0 were kept for following analyses.

### GWAS quality control

The common practice of GWAS QC is conducted through two dimensions of the data: samples and variants. All QC assessments and successive filtering were done using PLINK1.9^63^. Samples had more than 1% missing genotyped SNPs, different genetic sex with the record in the phenotypic database, abnormal autosomal heterozygosity were removed. Sex conflict and abnormal heterozygosity may be caused by DNA sample contamination. One of paired samples who have genetic relationship within three degree of relatedness was filtered out. SNPs had high rate of missing genotypes, and deviated from the Hardy–Weinberg equilibrium (HWE) test (*P* ≤ 1e−05) as well as SNPs on X and Y chromosomes and mitochondria were also removed. After variant QC, 5,169,391 common SNPs (MAF > 0.05) were including for the association analyses. PCA was performed using SNPs on autosomal chromosome by PLINK1.9^63^ to investigate population stratification. No clear sub-cluster was observed. Typical north to south Grandaunt was demonstrated by the first principal component (PC). Linear and logistic regression added top two PCs as covariates.

### Single-variant-based association

When phenotypes and genotypes have passed all the QC processes, the statistical analysis of disease associations can be performed. The basic analysis of genome-wide association data is a series of single-locus statistical tests, examining each SNP independently for association to the disease status^64^. The types of statistical tests utilized mostly depend on the types of disease phenotypes. For continuous phenotype, DOB values of the C13 test results, additive model into linear regression was performed in 224 cases to identify genes associated with severity of *H. pylori* infection. While for binary trait, the case/control status, additive model into logistic regression was utilized in 224 cases and 256 controls to search for genes related to susceptibility of *H. pylori*. According to the scree plot of PCA (Supplementary Fig. S2), the top 2 PCs could explain most of the variations of the population and no clear population stratification observed (Supplementary Fig. S2). After Wilcoxon test, there was no significant difference for age in case/control and cases. In the logistic regression model, *H. pylori* infection status ~ sex, we found there was no significant correlation between sex and *H. pylori* infection. Therefore, the association analyses were only adjusted the top 2 PCs. The regression model used in DOB values analysis was DOB values in cases ~ Genotype + PC1 + PC2. The regression model used in *H. pylori* infection was *H. pylori* status ~ Genotype + PC1 + PC2. The adaptive permutation tests were used to generate significance levels empirically. All the analyses were performed in PLINK1.9^63^. The Manhattan plots were generated in R 3.6.1. Regional plots were generated by LocusZoom tool^65^ (http://locuszoom.org/).

### Gene/pathway-based association

The standard analysis of genome-wide association study uses single SNP marker as the test unit. However, due to small sample size, the complex LD structures and ethnic differences among different populations, many replication studies have failed. To improve GWAS power, the gene-based association has been proposed^66^. The gene-based analysis combined the summary statistics of all variants within a putative gene (coding region and possible regulatory region) to obtain a single *P* value that represents the significance level of diseases and genes. Similarly, variants within genes on the same pathway were combined for pathway-based analysis. Gene-based and pathway-based analyses were performed using GCTA-fastBAT tool^67,68^. In gene-based analysis, a gene region was defined as ± 8 kb of UTRs of a gene.

### Metagenomics sequencing

Fresh stool samples were collected from recruited volunteers for metagenomics sequencing. The fecal DNA extractions were processed following the MetaHIT protocol, then single-end metagenomics sequencing was performed using BGISEQ-500 platform. The low-quality reads were discarded, and the host DNA were removed based on human genome reference (hg38) by SOAP2^69^ (version 2.22). Taxonomic analysis was performed using MetaPhlAn2^32^ following removal of human reads. The relative abundance of species and genera were used in the current study. Meanwhile, gene family abundance and pathway abundance of each sample were profiled using HUMAnN2^70^ with default parameters. HUMAnN2^70^ reported the abundance of gene families from the UniProt Reference Clusters (UniRef90), which were further mapped to microbial pathways from the MetaCyc metabolic pathway database.

Gut microorganisms belonging to Kingdom Bacteria were extracted for correlation analysis between *H. pylori* infection and microbial abundance. Bacteria were excluded if they have less than 10% carriers in the population. After filtering, the abundance of each bacterium was rescaled into percentage within an individual. Then, Wilcoxon test was used to compare the abundance of bacteria between *H. pylori* cases and controls. The *P* values at the same taxonomic levels were adjusted by FDR. Alpha diversity assessed the bacteria diversity within individuals was compared in different *H. pylori* infection status groups using Wilcoxon test. Beta diversity illustrating distance between individuals in terms of bacterial composition was demonstrated as principal coordinates analysis (PCoA) plot. Alpha and beta diversity analyses were conducted in R 3.6.2 with vegan^71^, phyloseq^72^ and microbiome packages^73^. Additionally, we dichotomized the abundance data to represent whether a bacterium is present in an individual. Logistic regression adjusted for age and sex was then used to analyze the associations between the presence of bacteria and the status of *H. pylori* infection. FDR method was used for multiple testing adjustment. In pathway analysis, pathways present in over 10% of samples were selected for subsequent analyses.

### Ethnics statement

The study protocol was reviewed and approved by the Institutional Review Board of BGI (No. BGI-IRB 16068). Review procedures in BGI-IRB meet GCP principles and relevant national regulations for this application. Written informed consent was obtained from all the study participants prior to their enrolment.

## Supplementary Information

Supplementary Information.

## Data Availability

The data supported the findings of this study has been deposited into CNGB Sequence Archive (CNGB)^74^ of China National GeneBank DataBase (CNGBdb)^75^ with accession number CNP0001557.

## References

[1] Hooi JK (2017). Global prevalence of *Helicobacter pylori* infection: Systematic review and meta-analysis. Gastroenterology.

[2] Montecucco C, Rappuoli R (2001). Living dangerously: How *Helicobacter pylori* survives in the human stomach. Nat. Rev. Mol. Cell Biol..

[3] Kusters JG, Van Vliet AH, Kuipers EJ (2006). Pathogenesis of *Helicobacter pylori* infection. Clin. Microbiol. Rev..

[4] Suerbaum S, Josenhans C (2007). *Helicobacter pylori* evolution and phenotypic diversification in a changing host. Nat. Rev. Microbiol..

[5] Berthenet E (2018). A GWAS on *Helicobacter pylori* strains points to genetic variants associated with gastric cancer risk. BMC Biol..

[6] Whitmire JM, Merrell DS (2019). *Helicobacter pylori* genetic polymorphisms in gastric disease development. Adv. Exp. Med. Biol..

[7] Graham DY (1991). Epidemiology of *Helicobacter pylori* in an asymptomatic population in the United States: Effect of age, race, and socioeconomic status. Gastroenterology.

[8] Malaty HM, Engstrand L, Pedersen NL, Graham DY (1994). *Helicobacter pylori* infection: Genetic and environmental influences: a study of twins. Ann. Intern. Med..

[9] Mayerle J (2013). Identification of genetic loci associated with *Helicobacter pylori* serologic status. JAMA.

[10] Hansson GK, Edfeldt K (2005). Toll to be paid at the gateway to the vessel wall. Arterioscler. Thromb. Vasc. Biol..

[11] Lagunes-Servin H (2013). Toll-like receptors and cytokines are upregulated during *Helicobacter pylori* infection in Children. Helicobacter.

[12] Simawaranon T, Wattanawongdon W, Tongtawee T (2017). Toll-like receptors are associated with *Helicobacter pylori* infection and gastric mucosa pathology. J. Clin. Microbiol..

[13] Falush D (2003). Traces of human migrations in *Helicobacter pylori* populations. Science.

[14] Ma J (2017). Associations between cytokine gene polymorphisms and susceptibility to *Helicobacter pylori* infection and *Helicobacter pylori* related gastric cancer, peptic ulcer disease: A meta-analysis. PLoS ONE.

[15] Negovan A, Iancu M, Fülöp E, Bănescu C (2019). *Helicobacter pylori* and cytokine gene variants as predictors of premalignant gastric lesions. World J. Gastroenterol..

[16] Wang D (2019). Alterations in the human gut microbiome associated with *Helicobacter pylori* infection. FEBS Open Bio.

[17] Gao J-J (2018). Association between gut microbiota and *Helicobacter pylori*-related gastric lesions in a high-risk population of gastric cancer. Front. Cell. Infect. Microbiol..

[18] Marshall CR (2008). Structural variation of chromosomes in autism spectrum disorder. Am. J. Hum. Genet..

[19] Kaminsky EB (2011). An evidence-based approach to establish the functional and clinical significance of copy number variants in intellectual and developmental disabilities. Genet. Med..

[20] Gao Y (2020). Long non-coding RNA *FGD5-AS1* regulates cancer cell proliferation and chemoresistance in gastric cancer through miR-153-3p/CITED2 axis. Front. Genet..

[21] Shimizu T (2014). Accumulation of somatic mutations in *TP53* in gastric epithelium with *Helicobacter pylori* infection. Gastroenterology.

[22] Chang Y-C (2008). Common variation in the fat mass and obesity-associated (*FTO*) gene confers risk of obesity and modulates BMI in the Chinese population. Diabetes.

[23] Xu X (2019). Relationship between *Helicobacter pylori* infection and obesity in Chinese adults: A systematic review with meta-analysis. PLoS ONE.

[24] Conteduca V (2013). *H. pylori *infection and gastric cancer: State of the art. Int. J. Oncol..

[25] Xu D (2017). *FTO* expression is associated with the occurrence of gastric cancer and prognosis. Oncol. Rep..

[26] Andersen KG (2012). Genome-wide scans provide evidence for positive selection of genes implicated in Lassa fever. Philos. Trans. R. Soc. Lond. B Biol. Sci..

[27] Mootha VK (2003). *PGC-1α*-responsive genes involved in oxidative phosphorylation are coordinately downregulated in human diabetes. Nat. Genet..

[28] Subramanian A (2005). Gene set enrichment analysis: A knowledge-based approach for interpreting genome-wide expression profiles. Proc. Natl. Acad. Sci. U.S.A..

[29] Guo Y (2020). Effect of *Helicobacter pylori* on gastrointestinal microbiota: A population-based study in Linqu, a high-risk area of gastric cancer. Gut.

[30] Guzman-Murillo MA, Ruiz-Bustos E, Ho B, Ascencio F (2001). Involvement of the heparan sulphate-binding proteins of *Helicobacter pylori* in its adherence to HeLa S3 and Kato III cell lines. J. Med. Microbiol..

[31] Epplein M (2011). *Helicobacter pylori* prevalence and circulating micronutrient levels in a low-income United States population. Cancer Prev. Res. (Phila.).

[32] Truong DT (2015). MetaPhlAn2 for enhanced metagenomic taxonomic profiling. Nat. Methods.

[33] Liu S (2019). Altered gut microbiota and short chain fatty acids in Chinese children with autism spectrum disorder. Sci. Rep..

[34] Zhai S (2019). Dietary butyrate suppresses inflammation through modulating gut microbiota in high-fat diet-fed mice. FEMS Microbiol. Lett..

[35] De Maesschalck C (2014). *Faecalicoccus acidiformans *gen. nov., sp. nov., isolated from the chicken caecum, and reclassification of *Streptococcus pleomorphus* (Barnes et al. 1977), *Eubacterium biforme* (Eggerth 1935) and *Eubacterium cylindroides *(Cato et al. 1974) as *Faecalicoccus pleomorphus* comb. nov., *Holdemanella biformis* gen. nov., comb. nov. and *Faecalitalea cylindroides* gen. nov., comb. nov., respectively, within the family *Erysipelotrichaceae*. Int. J. Syst. Evol. Microbiol..

[36] Kaakoush NO (2015). Insights into the role of *Erysipelotrichaceae* in the human host. Front. Cell. Infect. Microbiol..

[37] Brown K, DeCoffe D, Molcan E, Gibson DL (2012). Diet-induced dysbiosis of the intestinal microbiota and the effects on immunity and disease. Nutrients.

[38] Brahe L (2015). Specific gut microbiota features and metabolic markers in postmenopausal women with obesity. Nutr. Diabetes.

[39] Koh A, De Vadder F, Kovatcheva-Datchary P, Bäckhed F (2016). From dietary fiber to host physiology: Short-chain fatty acids as key bacterial metabolites. Cell.

[40] Wang L (2020). Acetic acid and butyric acid released in large intestine play different roles in the alleviation of constipation. J. Funct. Foods.

[41] Corvaglia L (1999). Accuracy of serology and 13C-urea breath test for detection of *Helicobacter pylori* in children. Pediatr. Infect. Dis. J..

[42] Hess ME, Brüning JC (2014). The fat mass and obesity-associated (*FTO*) gene: Obesity and beyond?. Biochim. Biophys. Acta Mol. Basis Dis..

[43] Loos RJ, Yeo GS (2014). The bigger picture of *FTO*—The first GWAS-identified obesity gene. Nat. Rev. Endocrinol..

[44] Arslan E, Atılgan H, Yavaşoğlu İ (2009). The prevalence of *Helicobacter pylori* in obese subjects. Eur. J. Intern. Med..

[45] Zhang J (2020). Persistent infection of *Helicobacter pylori* affects weight loss in obese population compared with persistent negative: A case-control study based on healthy Chinese. Helicobacter.

[46] Lin B, Qing X, Liao J, Zhuo K (2020). Role of protein glycosylation in host–pathogen interaction. Cells.

[47] Fransson LA (1987). Structure and function of cell-associated proteoglycans. Trends Biochem. Sci..

[48] Zhang T, de Waard AA, Wuhrer M, Spaapen RM (2019). The role of glycosphingolipids in immune cell functions. Front. Immunol..

[49] Mendz GL, Burns BP, Hazell SL (1995). Characterisation of glucose transport in *Helicobacter pylori*. Biochim. Biophys. Acta Gen. Subj..

[50] Mendz GL, Hazell SL, Burns BP (1994). The Entner–Doudoroff pathway in *Helicobacter pylori*. Arch. Biochem. Biophys..

[51] Mendz GL, Hazell SL, Burns BP (1993). Glucose utilization and lactate production by *Helicobacter pylori*. Microbiology.

[52] Marais A, Mendz GL, Hazell SL, Mégraud F (1999). Metabolism and genetics of *Helicobacter pylori*: The genome era. Microbiol. Mol. Biol. Rev..

[53] Som S (2015). Mechanisms linking metabolism of *Helicobacter pylori* to ^18^O and ^13^C-isotopes of human breath CO_2_. Sci. Rep..

[54] Liu Y (2019). Metabolic reprogramming results in abnormal glycolysis in gastric cancer: A review. Onco Targets Ther..

[55] Luo B (2016). ATP-dependent Lon protease contributes to *Helicobacter pylori*-induced gastric carcinogenesis. Neoplasia.

[56] Moyat M, Velin D (2014). Immune responses to *Helicobacter pylori* infection. World J. Gastroenterol..

[57] Li TH (2017). Alterations in gastric microbiota after *H. pylori* eradication and in different histological stages of gastric carcinogenesis. Sci. Rep..

[58] Durazzi F (2021). Comparison between 16S rRNA and shotgun sequencing data for the taxonomic characterization of the gut microbiota. Sci. Rep..

[59] Evrengul H (2007). Elevated homocysteine levels in patients with slow coronary flow: Relationship with *Helicobacter pylori* infection. Helicobacter.

[60] Rossi M, Amaretti A, Raimondi S (2011). Folate production by probiotic bacteria. Nutrients.

[61] Li H, Durbin R (2009). Fast and accurate short read alignment with Burrows–Wheeler transform. Bioinformatics.

[62] DePristo MA (2011). A framework for variation discovery and genotyping using next-generation DNA sequencing data. Nat. Genet..

[63] Purcell S (2007). PLINK: A tool set for whole-genome association and population-based linkage analyses. Am. J. Hum. Genet..

[64] Balding DJ (2006). A tutorial on statistical methods for population association studies. Nat. Rev. Genet..

[65] Pruim RJ (2010). LocusZoom: Regional visualization of genome-wide association scan results. Bioinformatics.

[66] Neale BM, Sham PC (2004). The future of association studies: Gene-based analysis and replication. Am. J. Hum. Genet..

[67] Yang J, Lee SH, Goddard ME, Visscher PM (2011). GCTA: A tool for genome-wide complex trait analysis. Am. J. Hum. Genet..

[68] Bakshi A (2016). Fast set-based association analysis using summary data from GWAS identifies novel gene loci for human complex traits. Sci. Rep..

[69] Li R (2009). SOAP2: An improved ultrafast tool for short read alignment. Bioinformatics.

[70] Franzosa EA (2018). Species-level functional profiling of metagenomes and metatranscriptomes. Nat. Methods.

[71] Oksanen, J. *et al.* vegan: Community Ecology Package. *R package version 2.5-7*, https://CRAN.R-project.org/package=vegan (2020).

[72] McMurdie PJ, Holmes S (2013). phyloseq: An R package for reproducible interactive analysis and graphics of microbiome census data. PLoS ONE.

[73] Lahti, L. & Shetty, S. Tools for microbiome analysis in R. *Version*, http://microbiome.github.com/microbiome (2017).

[74] Guo X (2020). CNSA: A data repository for archiving omics data. Database (Oxford).

[75] Chen FZ (2020). CNGBdb: China National GeneBank DataBase. Yi Chuan.

