# Supplementary material for: Glycosaminoglycan biosynthesis pathway in host genome is associated with Helicobacter pylori infection
Source: Sci Rep. 2021 Sep 14;11:18235. doi: 10.1038/s41598-021-97790-7 (PMC8440747; doi:10.1038/s41598-021-97790-7)
Supplement: Supplementary file 1 — Supplementary Information. [file 41598_2021_97790_MOESM1_ESM.zip › Supplementary Figures.docx]

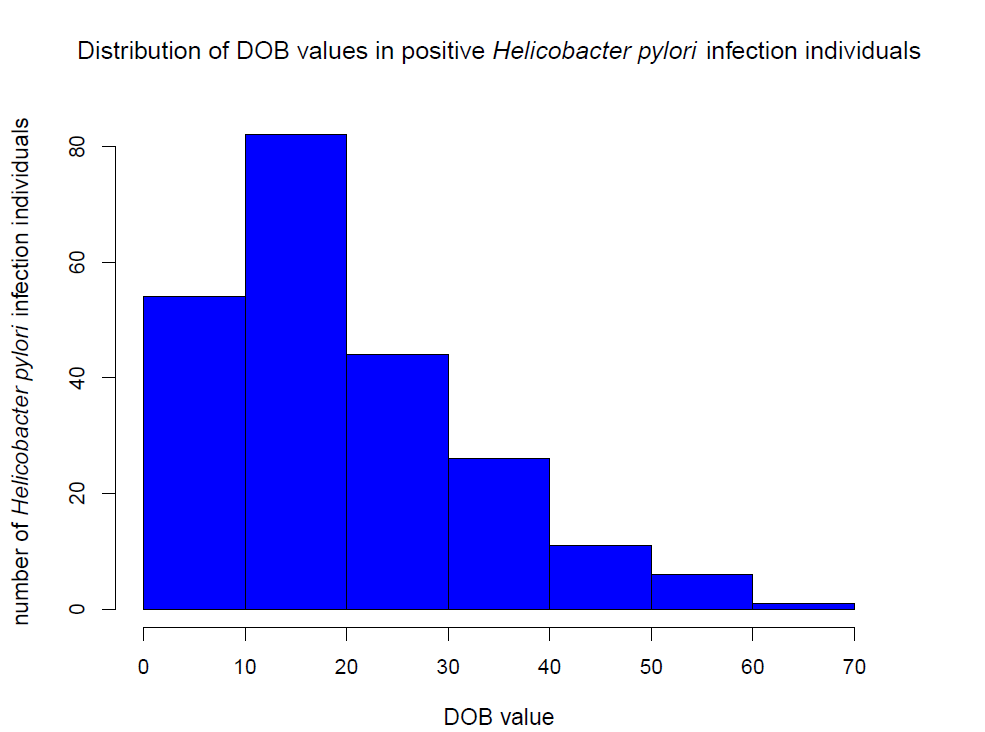
**Supplementary Fig S1.** Distribution of DOB value of *Helicobacter pylori* infection. DOB value: Delta Over Baseline (DOB), which is the difference between post-drug δ value minus pre-drug δ value. δ value, which measured the ^13^C/^12^C ratio in the breath samples.


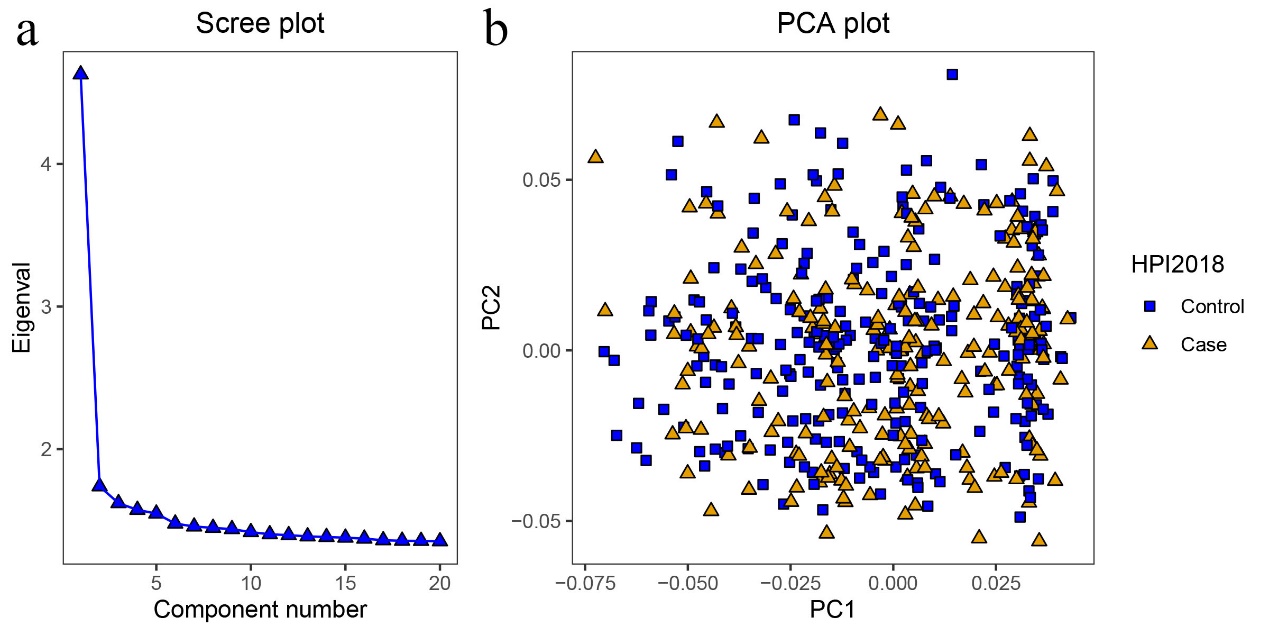


**Supplementary Fig. S2.** Plots for the principal components analysis (PCA) result. (a): Scree plot for principal components analysis result; (b): PCA plot for the top 2 principal components.


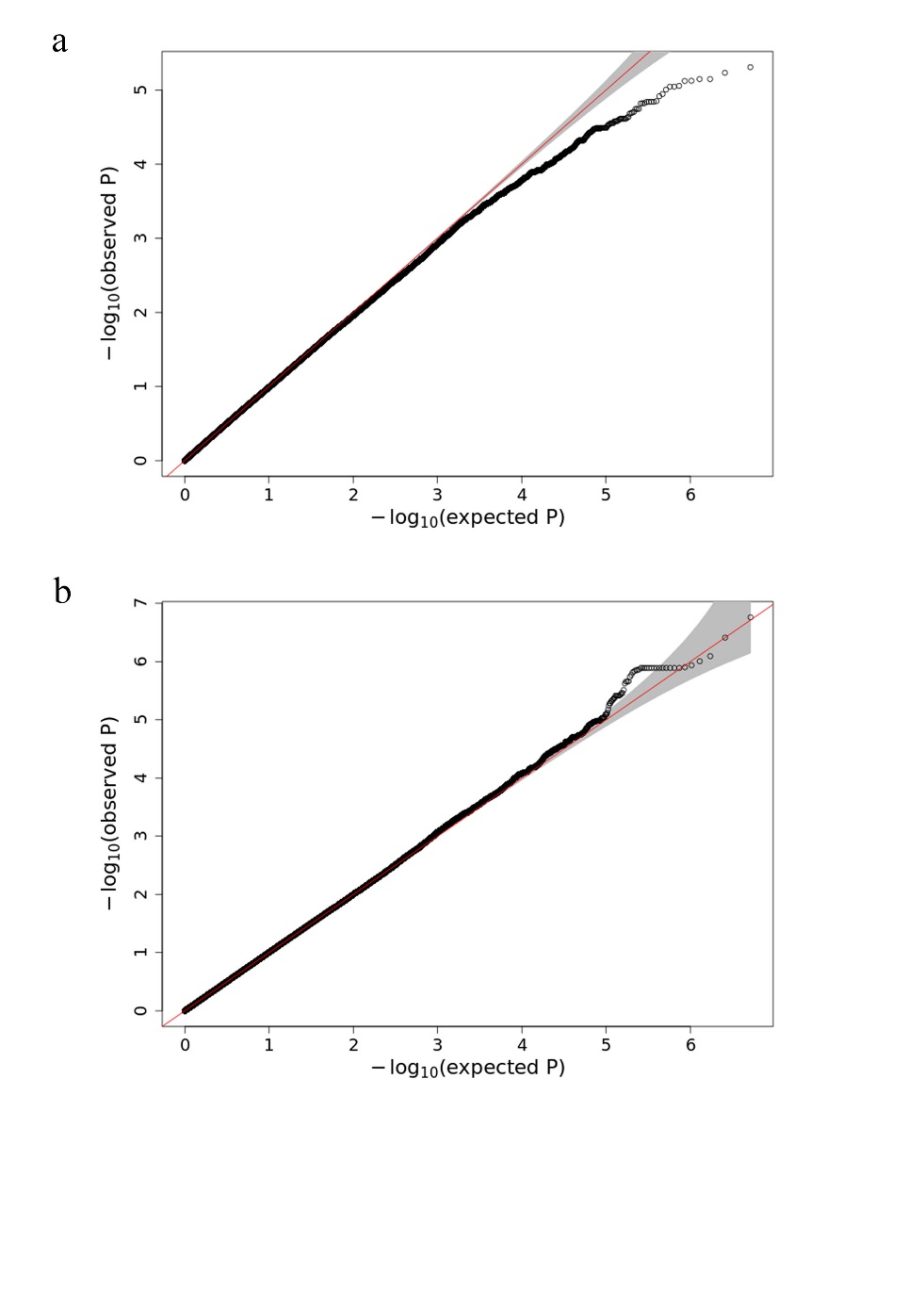


**Supplementary Fig. S3.** QQ-plots of the GWAS results based on on case/control association (a) and DOB value for cases association (b). QQ-plots comparing the expected (under the null hypothesis) and actual (observed) -log10 p-values from the GWAS results adjusted for the top two principal components of the *Helicobacter pylori* infection cases versus all controls (a), and the urea measurements in *Helicobacter pylori* infection cases (b).


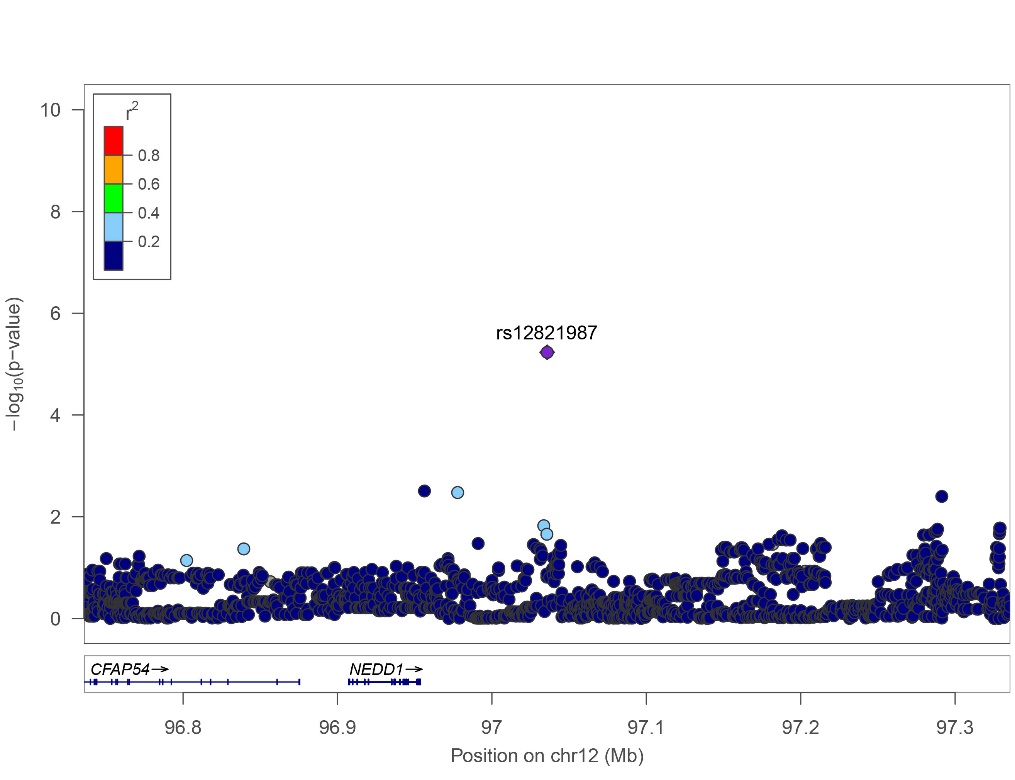


**Supplementary Fig. S4.** *Helicobacter pylori* infection associated genes identified from logistic regression-based GWAS results. Regional association plot for the rs12821987 based on the GWAS results from the *Helicobacter pylori* infection cases versus controls. The color of the variants is based on the linkage disequilibrium with rs12821987. For all plots, each point represents a SNP, where the x axis represents the position of the SNPs and the y axis the -log10 p values of the GWAS results. Each point is color-coded with the r^2^ value as calculated with the source of LD information retrieved from hg38/1000 Genomes Nov 2014 EAS (Asian). Plot shows the most significant SNPs flank 300 kb.


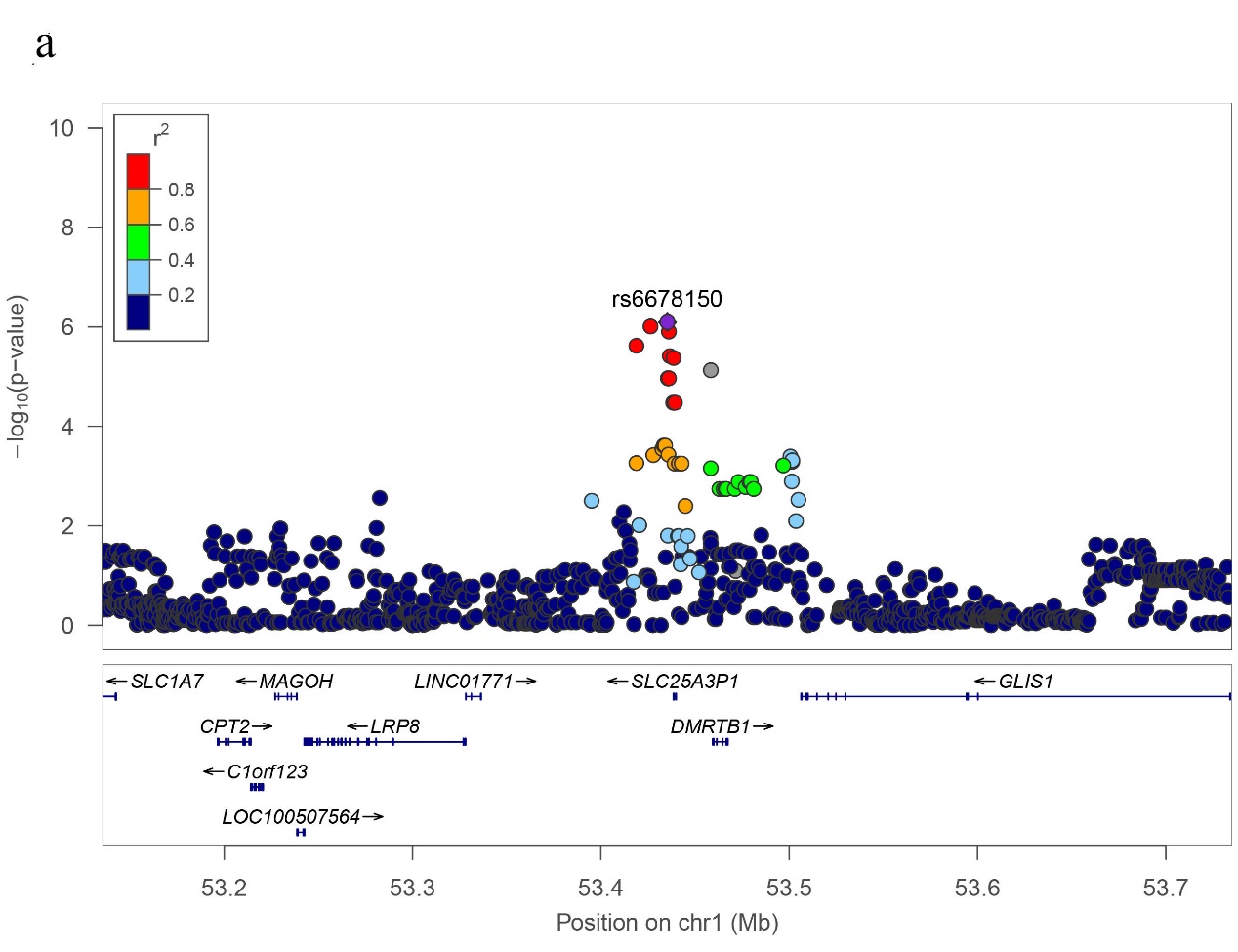


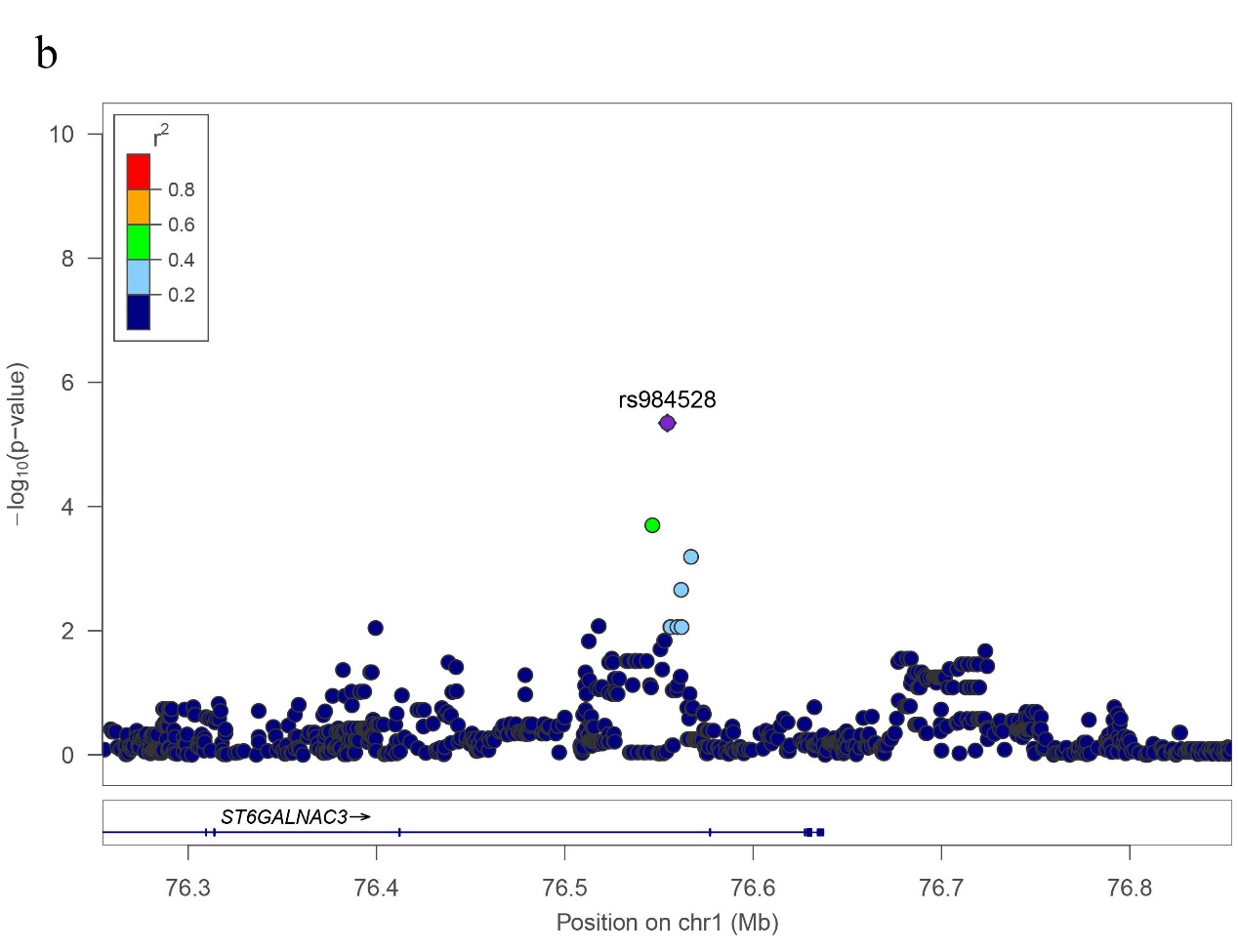


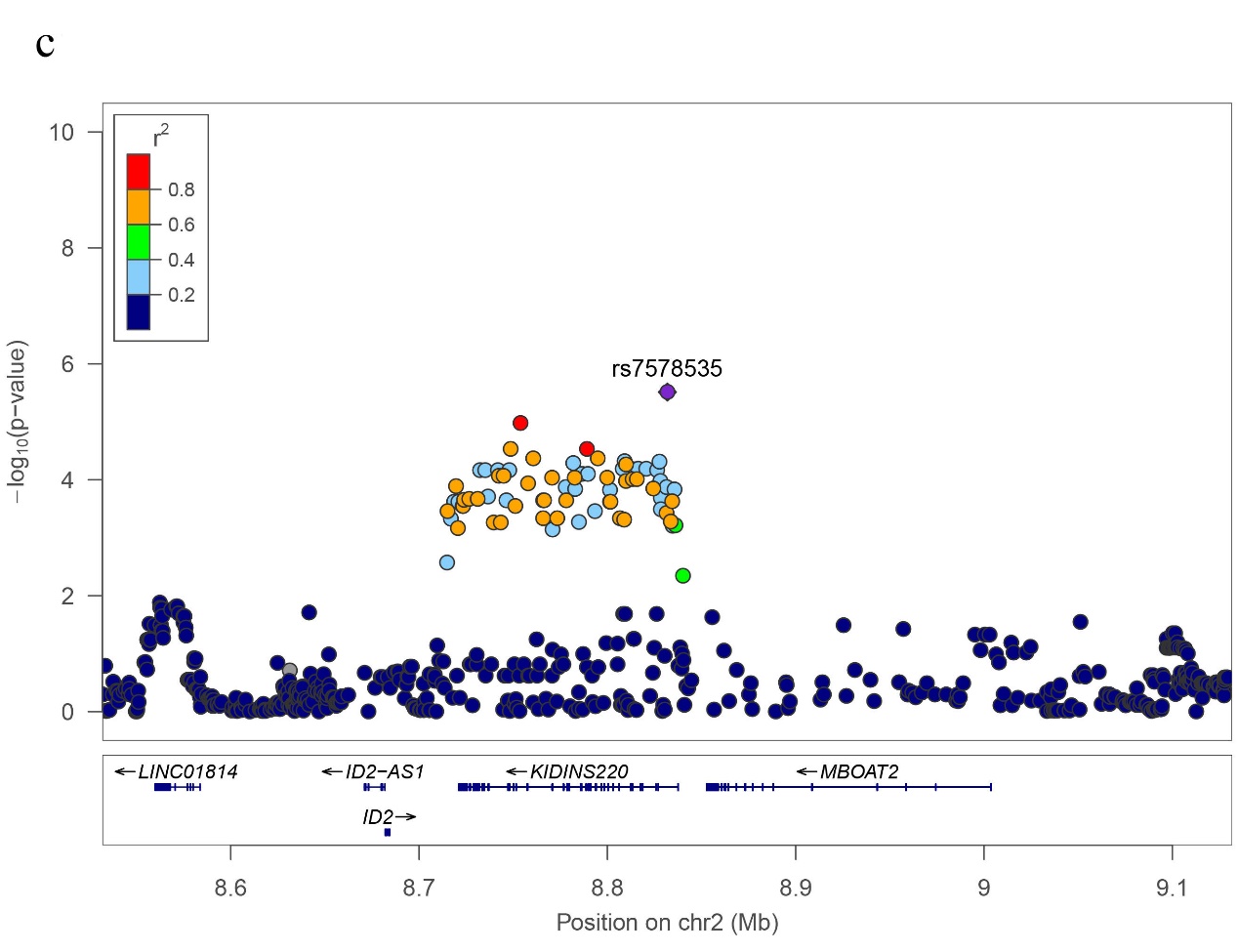


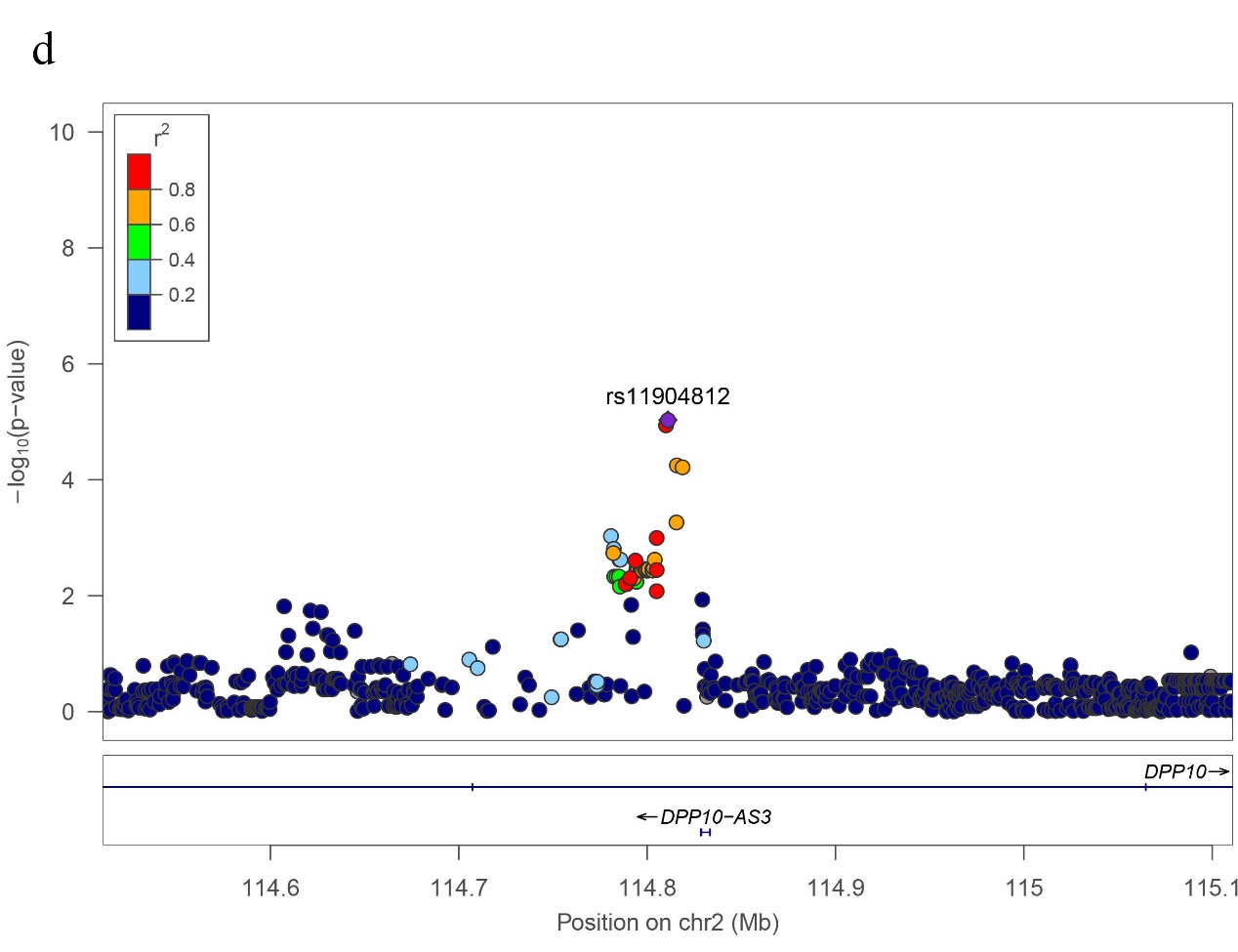


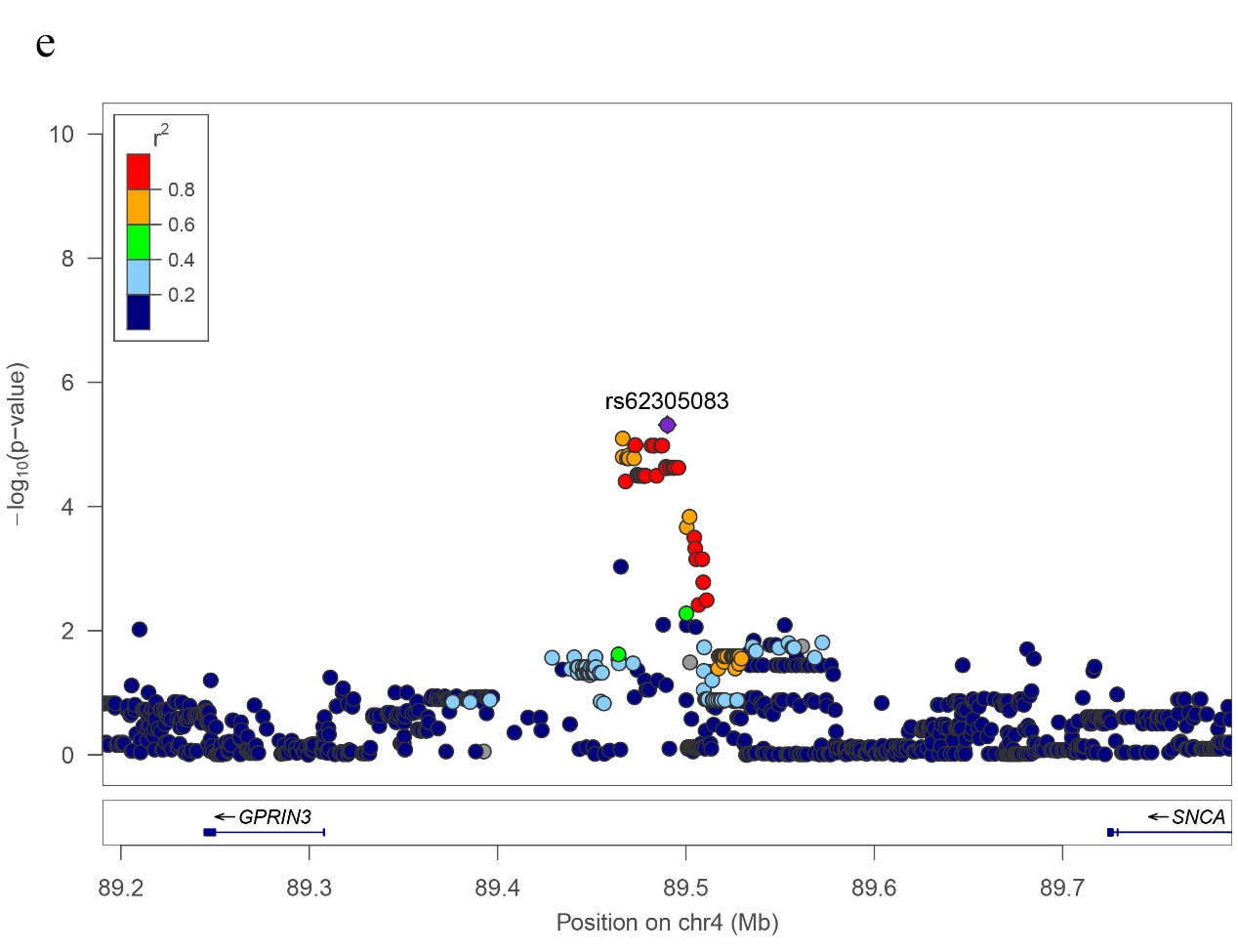

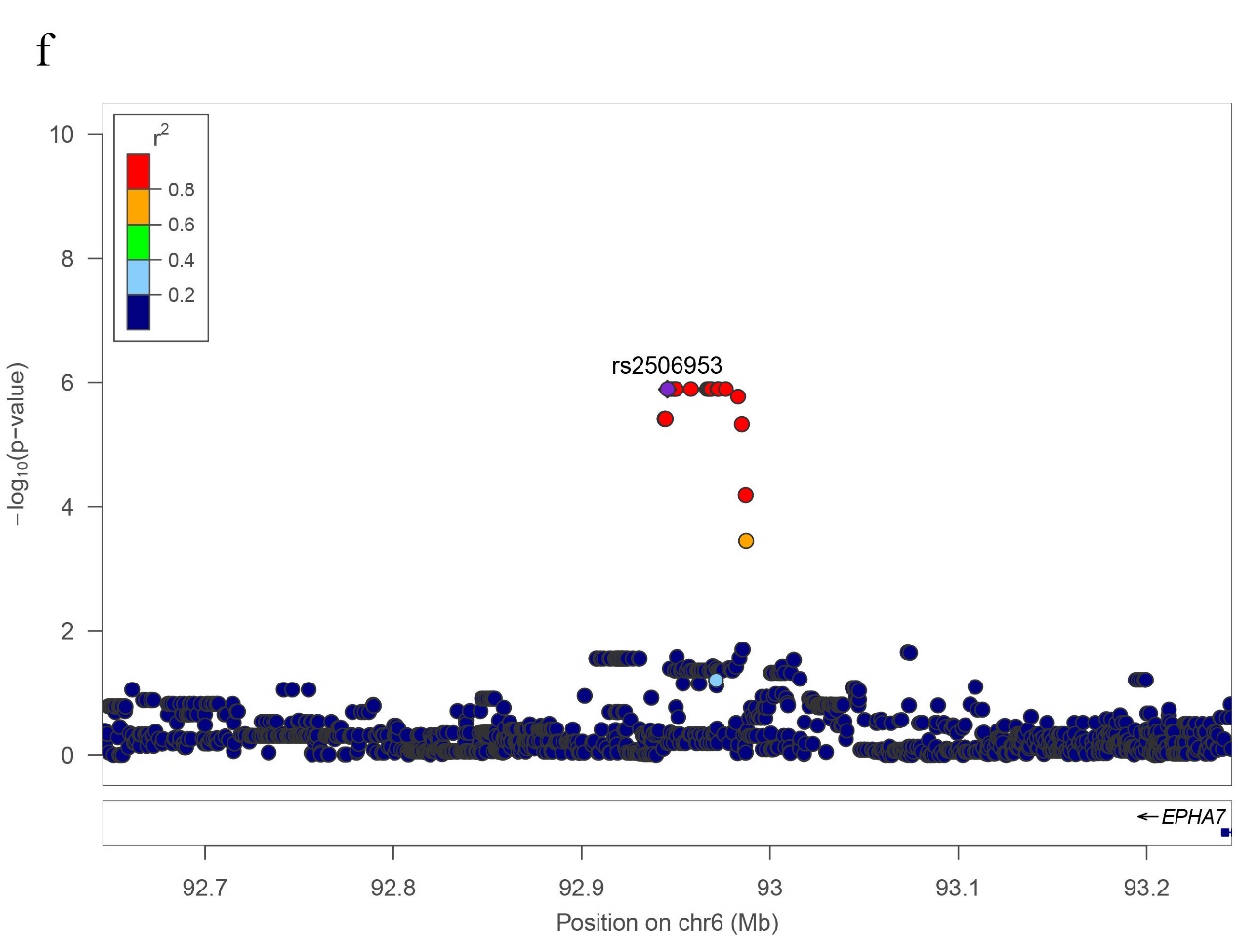


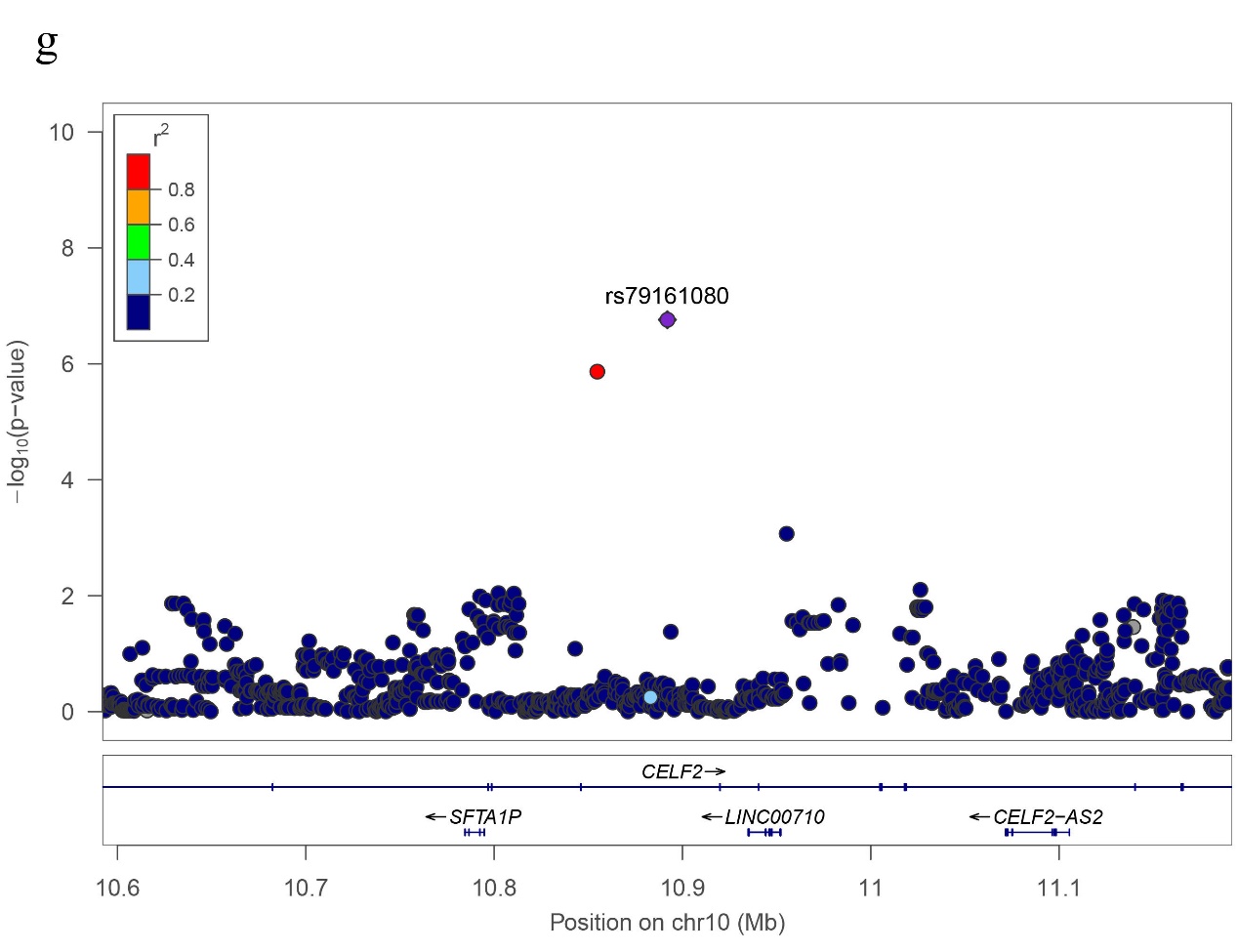


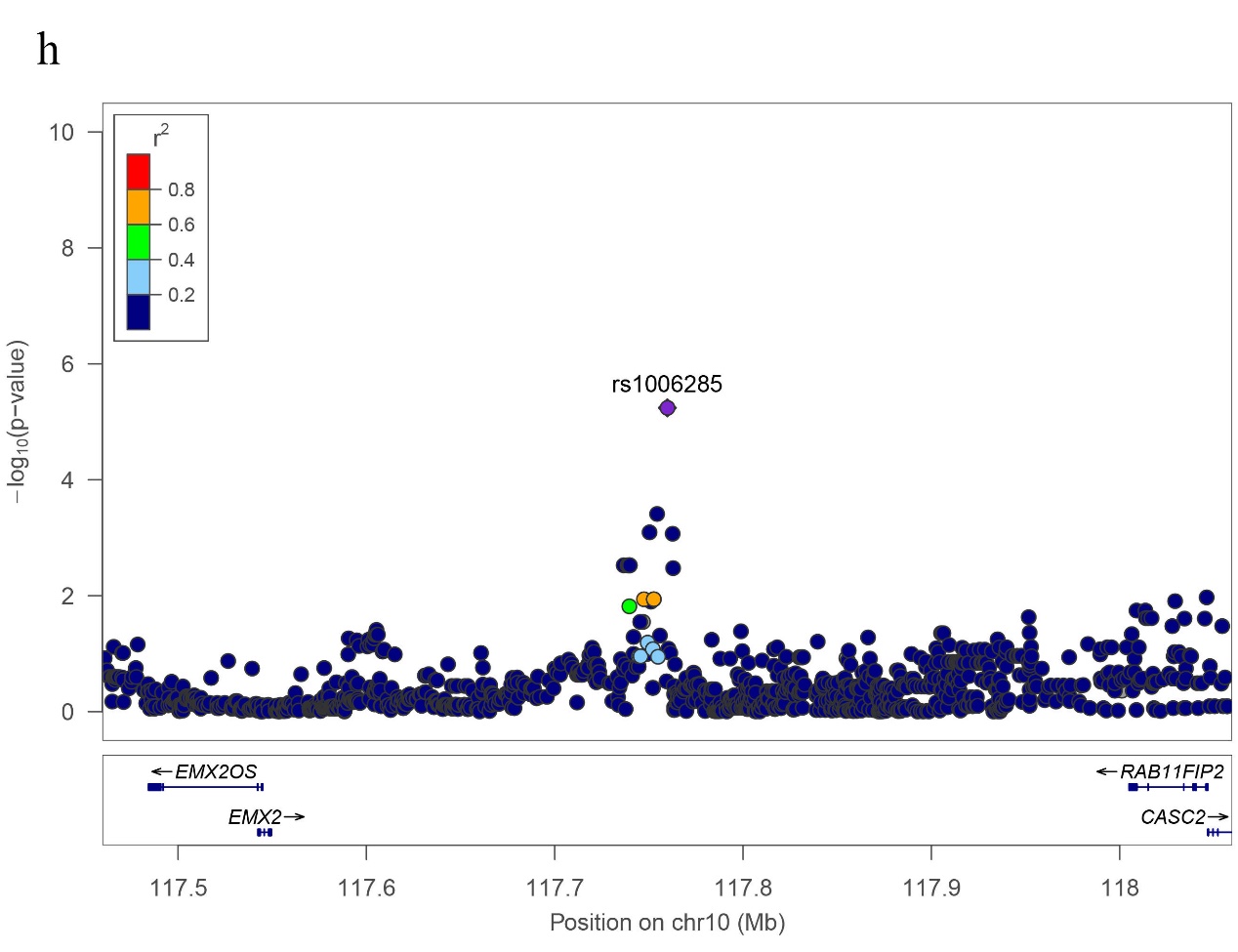

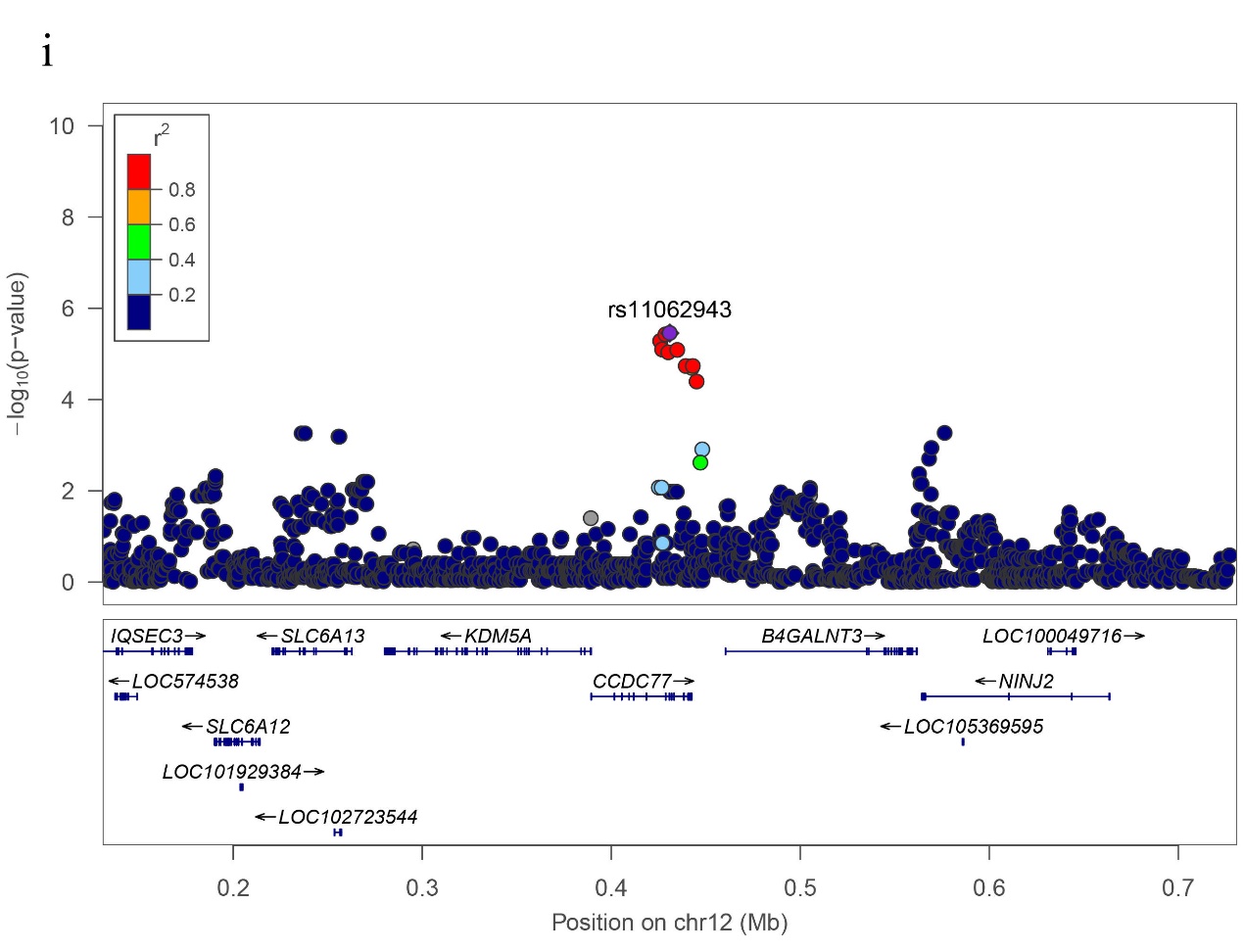

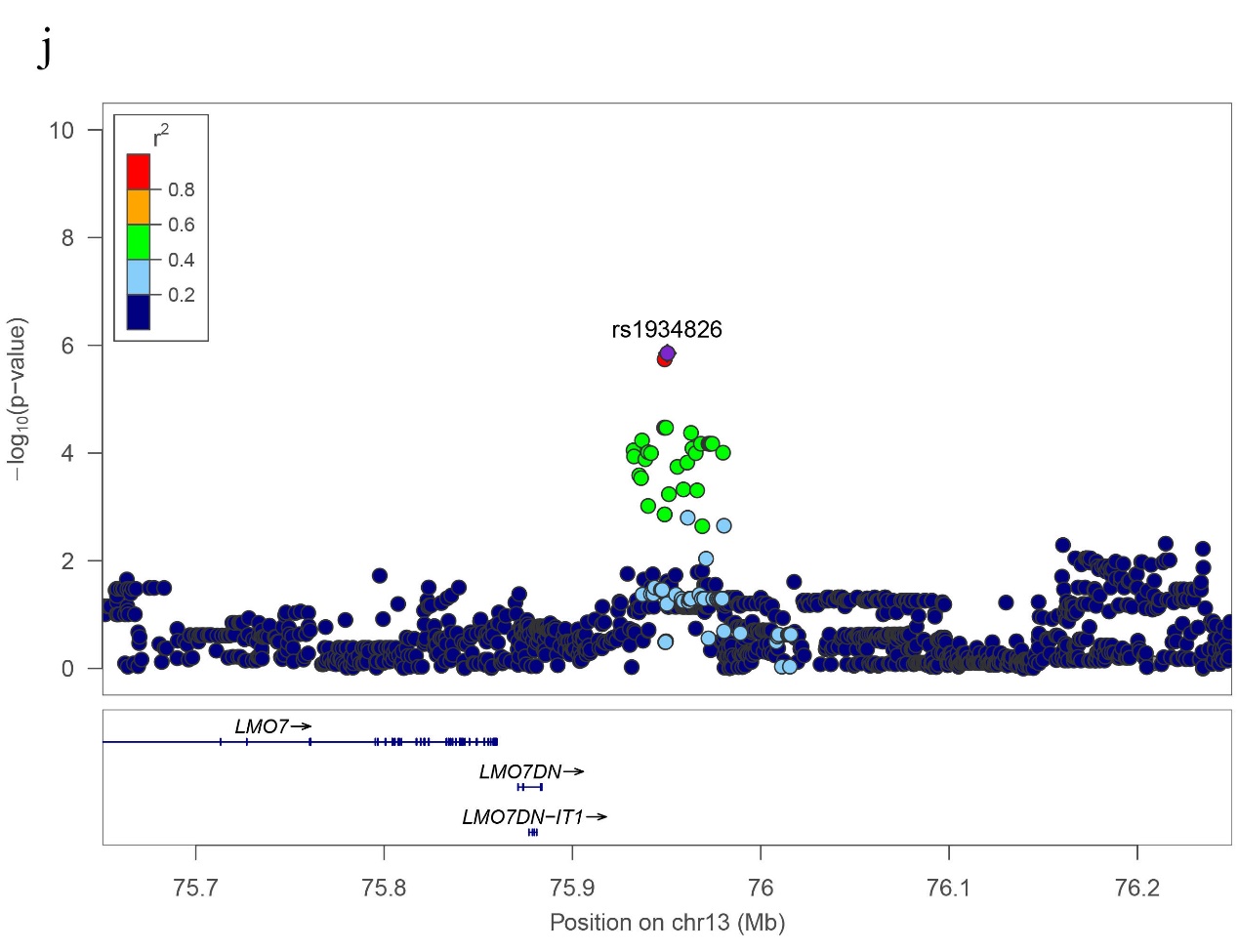


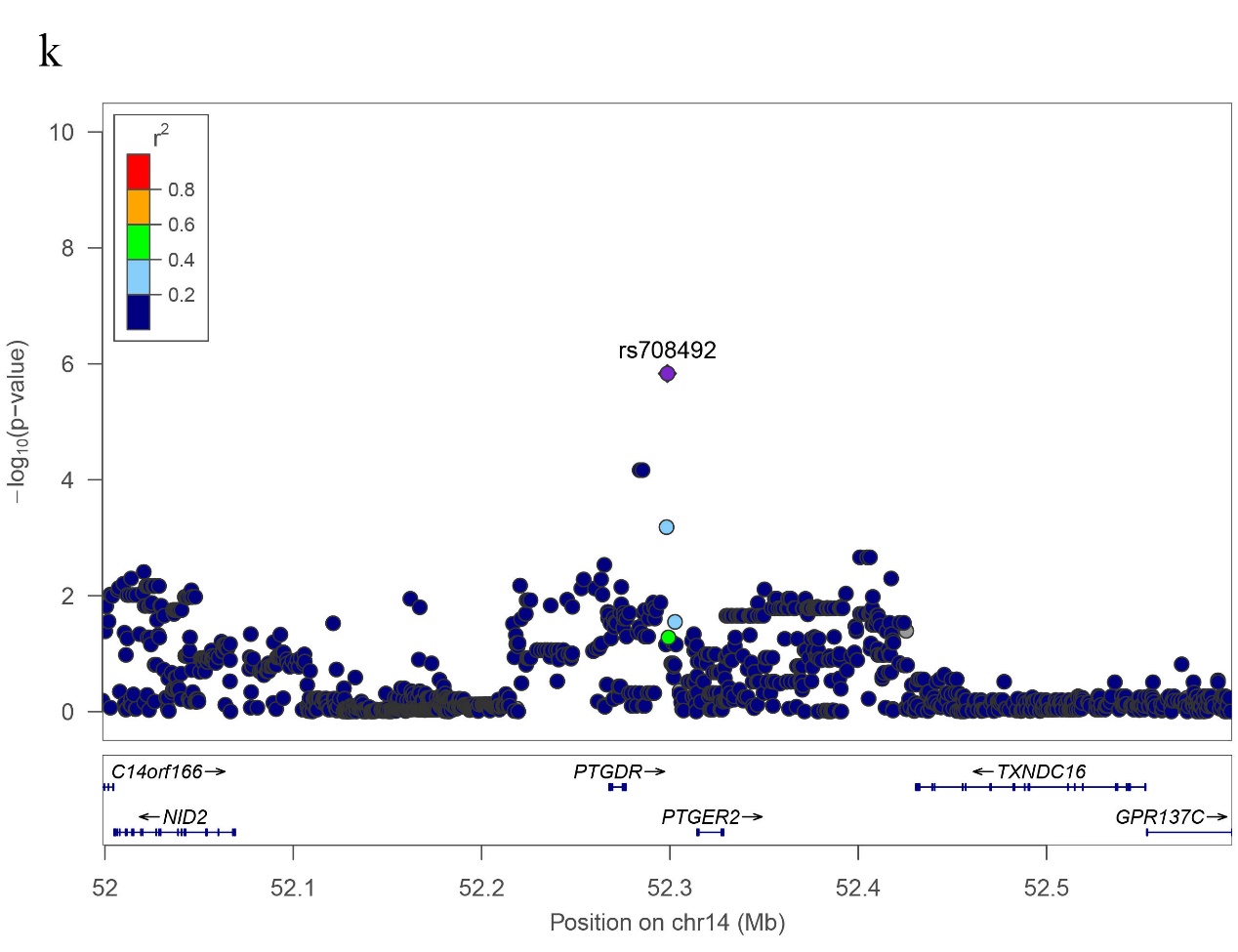


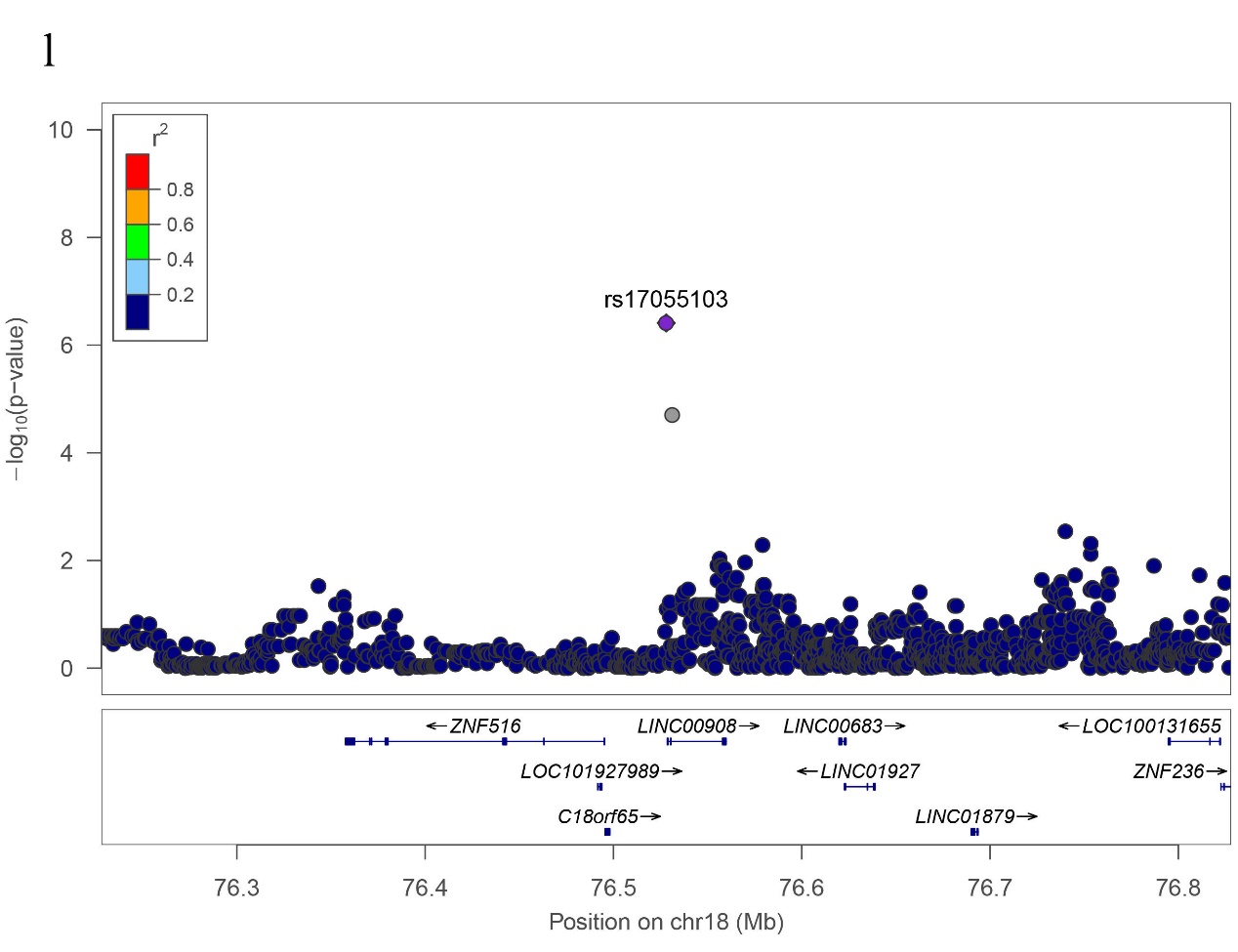


**Supplementary Fig. S5.** *Helicobacter pylori* infection associated genes identified from linear regression-based GWAS result. Regional association plots for the regions containing associated genes based on the GWAS results from the *Helicobacter pylori* infection cases. Regional association plots for the rs6678150 (*SLC25A3P1*) (a), rs984528 (*ST6GALNAC3*) (b), rs7578535 (*KIDINS220*) (c), rs11904812 (*DPP10*) (d), rs62305083 (intergenic) (e), rs2506953 (upstream *EPHA7*) (f)*,* rs79161080 (*CELF2*) (g)*,* rs1006285 (intergenic) (h)*,* rs11062943 (*CCDC77*) (i), rs1934826 (downstream *LMO7DN*) (j), rs708492 (intergenic) (k), rs17055103 (intergenic) (l). The color of the variants is based on the linkage disequilibrium with top SNP for each region. For all plots, each point represents a SNP, where the x axis represents the position of the SNPs and the y axis the -log10 p values of the GWAS results. Each point is color-coded with the r^2^ value as calculated with the source of LD information retrieved from hg38/1000 Genomes Nov 2014 EAS (Asian). Plots show the most significant SNPs flank 300 kb.
